# Antimicrobial resistance in *Bordetella pertussis*: A systematic review and meta-analysis

**DOI:** 10.1017/S0950268826101010

**Published:** 2026-02-06

**Authors:** Keer Ma, Wuming Sun, Gujie Pan, Yongjiang Xu, Yuan Shi, Yao Chen

**Affiliations:** 1 Department of Respiratory Diseases, Shaoxing Central Hospital, Zhejiang, China; 2 The Central Affiliated Hospital, Shaoxing University, Zhejiang, China

**Keywords:** antimicrobial resistance, *Bordetella pertussis*, macrolides, minimum inhibitory concentration, whooping cough

## Abstract

Since the first report of erythromycin-resistant *Bordetella pertussis* (*B. pertussis*) in Arizona in 1994, macrolide-resistant strains have emerged worldwide, threatening pertussis control. This systematic review and meta-analysis aimed to quantify the prevalence and temporal trends of this resistance. Four databases (PubMed, Embase, Cochrane Library, Web of Science) were searched for studies on *B. pertussis* antimicrobial susceptibility through December 2024. Among 57 included studies (1994–2024), pooled resistance rates (breakpoint ≥32 mg/L) were: erythromycin 21% (95% CI 11–32%), azithromycin 25% (95% CI 12–40%), clarithromycin 15% (95% CI 4–30%), and clindamycin 49% (95% CI 28–70%). Subgroup analyses by country, year, and test method are presented. No trimethoprim/sulfamethoxazole (STX) resistance was reported. Six Japanese isolates showed high-level nalidixic acid resistance (MIC >256 mg/L). Seventy-seven studies contributed to MIC90 data for carbapenems, tetracyclines, aminoglycosides, quinolones, macrolides, cephalosporins, and others. Selected penicillins and intravenous third-generation cephalosporins demonstrated strong in vitro activity, suggesting alternative treatment options. Macrolide-resistant *B. pertussis* has increased significantly over the past decade. Due to the high burden of antibiotic resistance in China, enhanced surveillance is warranted, while continued monitoring in other countries also remains necessary.

## Introduction

Pertussis, commonly known as whooping cough, is an acute respiratory illness in humans caused by the Gram-negative bacterium *Bordetella pertussis* (*B. pertussis*) [1]. The disease is characterized primarily by severe paroxysmal coughing, often accompanied by classic symptoms such as an inspiratory whoop and post-tussive vomiting [2]. Whooping cough is highly contagious, whose basic reproduction number (R0) is about 5.5 [3], nearly four times more than that of influenza (R0 = 1.28) [4]. In the pre-vaccine era, the vast majority of children were susceptible to pertussis. Although the incidence of pertussis declined dramatically following the widespread implementation of vaccination programmes in the mid-20th century [5], the incidence of reported pertussis in many countries progressively increased in recent decades, which is called ‘recurrence of whooping cough’ [6, 7]. At the same time, in recent years, several countries have reported the incidence of disease in adolescents and adults [8, 9]. Thus, the situation of whooping cough demands research attention.

The first isolation of *B. pertussis* was reported by Bordet and Gengou in 1906 [10]. Since then, this microorganism has been widely studied by scholars. *B. pertussis*, a Gram-negative bacillus of the *Bordetella* genus, is a fastidious microorganism with specific and demanding nutritional requirements. In addition, *B. pertussis* grows slowly and often takes up to 5 to 7 days [11], limiting the timeliness for acute management and leading to difficulties in routine clinical testing. Antibiotics are believed to be most effective when administered early during the illness. Macrolides are the treatment of choice for *B. pertussis* [12]. In the United States, erythromycin has been used for more than 65 years to treat patients [13]. However, since the first report of erythromycin-resistant *B. pertussis* in Arizona in 1994 [14], more drug-resistant strains have been discovered worldwide, especially in China during recent years, which warrants attention. Currently, a few studies have summarized resistance data and minimum inhibitory concentration (MIC) on *B. pertussis*, and no associated meta-analysis was found. In this study, we aimed to review the data on the resistance of antimicrobials to *B. pertussis* and the MIC_90_ (90% minimum inhibitory concentration) of different kinds of antibiotics. These data serve as a guide for adjusting the antibiotic therapy for whooping cough.

## Method

### Search strategy and study selection

This systematic review and meta-analysis was conducted based on the Preferred Reporting Items for Systematic Reviews and Meta-Analysis (PRISMA) guidelines. The protocol of this systematic review has been registered in PROSPERO (ID: CRD42022325142). A comprehensive literature search was conducted across four electronic databases (PubMed, Embase, Cochrane Library, and Web of Science) from inception until December 2024, using the following search terms: (“Pertussis” OR “Pertusses” OR “Whooping Cough”) AND (“Antimicrobial-Drug Resistance” OR “drug resistance” OR “antibiotic resistance” OR “erythromycin” OR “beta-lactams” OR “Clarithromycin” OR “cephalosporins” OR “meropenem” OR “clindamycin” OR “tetracyclines” OR “quinolones” OR “macrolides” OR “carbapenems”) in all fields. No language restrictions were used in the search strategy. However, the criterion for inclusion was that studies must have an abstract available in English. All the records were merged and imported into EndNote X7 (Thomson Reuters, New York, NY, USA) to remove duplicates. Rayyan (https://www.rayyan.ai/) was used for screening and marking the records.

### Data selection criteria and quality assessment

Study selection was performed independently by two reviewers (Keer Ma and Yao Chen). Studies were excluded for the following reasons: (1) absence or unclear reporting of antibiotic resistance data; (2) non-original research (*e.g.* reviews, meta-analyses, conference abstracts) or studies focussing on drug metabolism; (3) unavailability of full text; (4) sample size with fewer than five isolates or the use of non-human clinical strains; (5) duplication of previously reported data. Any inconsistencies with the selection of the article were resolved by a third author (Gujie Pan) based on the criteria above to reduce mistakes.

The adapted Newcastle–Ottawa Scale (Supplementary Table 1) was used by two independent authors (Keer Ma and Yao Chen) to assess the methodological quality of the included cross-sectional studies [15, 16]. Based on their scores across the domains of selection, comparability, and outcome assessment, studies were categorized as of high (≥5 points), moderate (3–4 points), or low (≤2 points) quality. A higher score indicated a higher study quality. Any cases with a disagreement were resolved through discussion. A third reviewer (Gujie Pan) adjudicated in any cases where there was a disagreement.

### Data extraction

The following data were extracted from each included study: (1) first author; (2) publication year; (3) year of isolate collection; (4) study country; (5) total number of isolates; (6) antimicrobial susceptibility testing (AST) method; (7) interpretation of resistance; (8) number of resistant isolates; and (9) MIC_90_ values.

### Definition of resistance

Neither the Clinical and Laboratory Standards Institute (CLSI) nor the European Committee on Antimicrobial Susceptibility Testing (EUCAST) has defined specific susceptibility and resistance breakpoints for *B. pertussis.* In accordance with relevant literature, an MIC of ≥32 mg/mL was used to define the resistance of erythromycin, azithromycin, clarithromycin, and clindamycin. Other antibiotics were evaluated for determining susceptibility by using the breakpoints specified by the 35th edition of the CLSI for *Haemophilus influenzae.*

### Data analysis

The pooled resistance rate (PRR) for each antimicrobial was derived using a random-effects model to address anticipated heterogeneity. All statistical analyses were conducted with R software (version 4.0.3). Heterogeneity was quantified using the *I^2^* statistic [17]. The existence of publication bias was assessed by funnel plots and Egger’s test. We performed subgroup analyses to assess the influence of publication year, country, and AST method on the PRR. All statistical results are reported as point estimates with 95% confidence intervals (CIs).

## Results

### Search results

**Figure 1.**
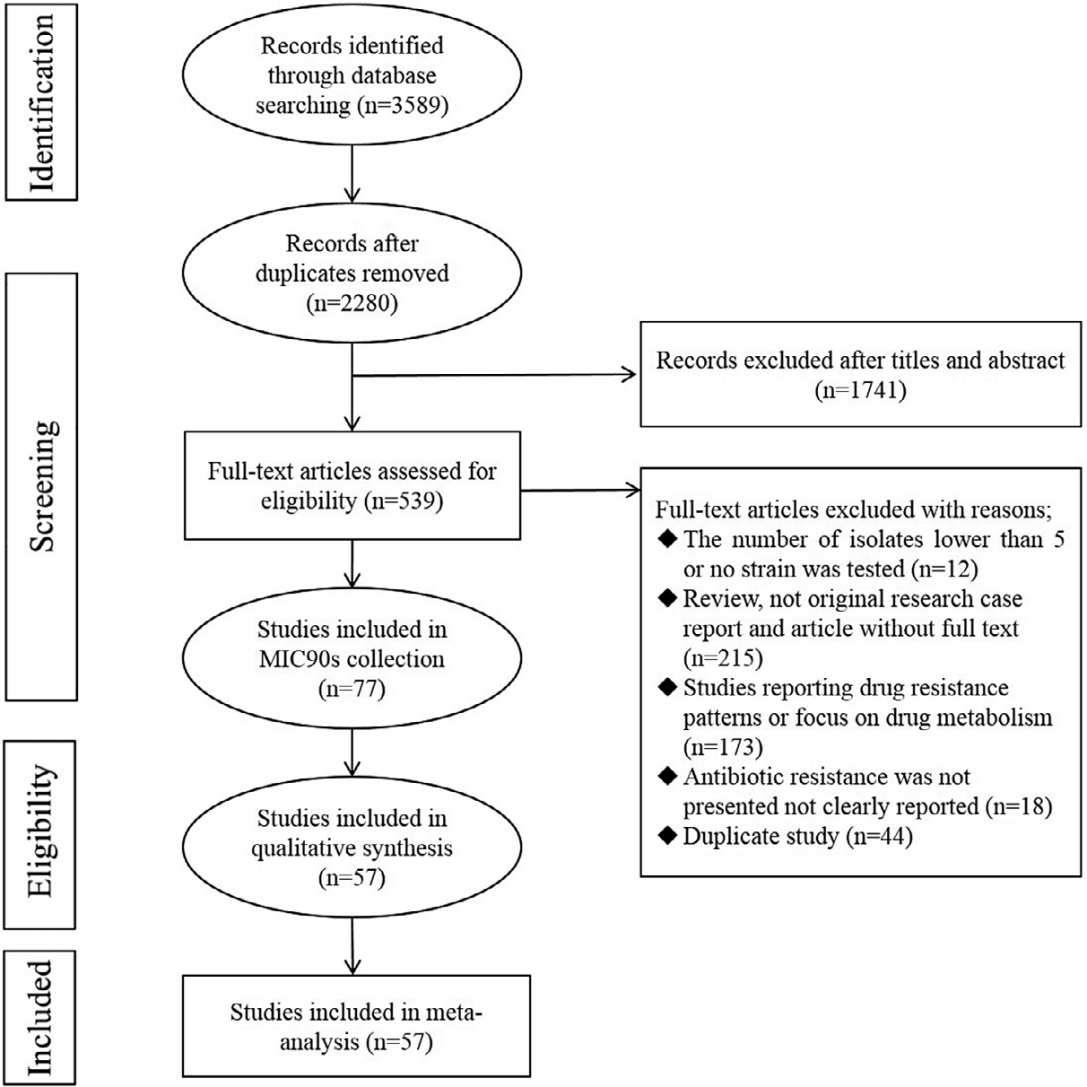
Flow diagram showing the study selection process.

As shown in Figure 1, 3,589 articles were collected through four electronic databases published until December 2024. After removing duplication by using Endnote X7, 2,280 records remained. After an initial screening of the title and abstract, 1,741 articles were excluded due to their irrelevance, and the full texts of the remaining 539 articles were reviewed. At the end of the screening, 77 eligible articles were included in this review to extract MIC_90_ values, and 57 studies published from 1994 to 2024 were included in the meta-analysis according to the preset inclusion and exclusion criteria.

### Antimicrobial resistance analysis

#### Characteristics of the included studies

The 57 included studies describing the drug resistance of *B. pertussis* were selected from 16 countries covered 5,868 isolates. Most of the studies originated in China (*n* = 23), followed by the United States (*n* = 11). Epsilometer test (ET) (*n* = 40) was the most frequent AST method used, followed by agar dilution (AD, *n* = 16). All studies had a cross-sectional design, and the mean Newcastle–Ottawa score was 4.86. The quality was high in 36 (63.2%) studies and medium in 21 (36.8%) studies. All 57 studies in the meta-analysis reported resistance to macrolides (azithromycin, erythromycin, clarithromycin) and clindamycin (Table 1).

**Table 1.** Extracted information from eligible studies included in the meta-analysis during 1994–2024

ID	Author	Published year	Year of isolate	Country of study	Testing method	Number of isolates	Resistance no. (%)			
							Azithromycin	Erythromycin	Clarithromycin	Clindamycin
1	Lewis K	1995	1994	USA	ET	8	ND	1(12.5)	1(12.5)	1(12.5)
2	Horikawa K	1995	1990–1993	Japan	AD	27	ND	0(0)	ND	ND
3	Aoyama T	1996	1990–1995	Japan	AD	60	0(0)	0(0)	0(0)	ND
4	Cimolai N	1997	1993–1996	Canada	AD	66	ND	0(0)	ND	ND
5	Korgenski EK	1997	1985–1997	USA	ET	47	ND	1(2.1)*	ND	ND
6	Hoppe JE	1998	1994–1997	Germany	AD	34	0(0)	0(0)	0(0)	ND
7	Brett M	1998	1991–1995	New Zealand	AD	88	ND	0(0)	ND	ND
8	Mortensen JE	2000	1987–1997	USA	AD	102	0(0)	0(0)	ND	ND
9	Hill BC	2000	NM	USA	AD	52	ND	1(1.9)	ND	ND
10	Gordon KA	2001	1998–1999	USA	ET	36	0(0)	0(0)	0(0)	0(0)
11	Chodorowska M	2001	1968, 1997–1999	Poland	AD	55	0(0)	0(0)	0(0)	ND
12	Bartkus JM	2003	1997–1999	USA	ET	577	5(0.9)	5(0.9)	5(0.9)	ND
13	Yamaguchi K	2003	NM	Japan	AD	35	ND	0(0)	ND	ND
14	Bourgeois N	2003	NM	USA	AD	51	ND	12(23.5)**	ND	ND
15	Ohtsuka M	2004	2001–2002	Japan	ET	26	0(0)	0(0)	0(0)	0(0)
16	Galanakis E	2006	2002–2006	USA	ET	100	0(0)	0(0)	ND	0(0)
17	Sintchenko V	2007	1971–1978,1995–2000,2001–2006	Australia	AD	99	ND	0(0)	ND	ND
18	Yao SM	2008	2003–2007	China	ET	28	0(0)	0(0)	0(0)	ND
19	Ohtsuka M	2009	2004–2006	Japan	ET	14	ND	0(0)	ND	ND
20	Fry NK	2010	2001–2009	England	ET	583	0(0)	0(0)	0(0)	ND
21	Luo J	2014	2013–2014	China	ET	7	5(71.4)	5(71.4)	5(71.4)	5(71.4)
22	Horiba K	2014	2008–2012	Japan	ET	30	0(0)	0(0)	0(0)	ND
23	Spicer KB	2014	2010–2011	Columbus	ET	15	0(0)	0(0)	0(0)	ND
24	Shahcheraghi F	2014	2009–2010	Iran	AD	11	0(0)	2(18.2)**	2(18.2)	ND
25	Theofiles AG	2014	2012	USA	ET	12	ND	0(0)	ND	ND
26	Marchand-Austin A	2014	2011–2012	Canada	ET	275	0(0)	ND	ND	ND
27	Yang Y	2015	1970s,2000–2008,2013–2014	China	ET	124	91(71.7)	91(71.7)	91(71.7)	91(71.7)
28	Mirzaei B	2015	2009–2010	Iran	AD	11	0(0)	1(9.1)**	1(9.1)	ND
29	Li Y	2015	2012–2013	China	ET	16	14(87.5)	14(87.5)	ND	ND
30	Hardy DJ	2016	2010–2014	USA	AD	34	0(0)	ND	0(0)	ND
31	Jakubů V	2017	1967–1999,2004–2010,2011–2015	Czech	AD	135	0(0)	0(0)	0(0)	ND
32	Stefanelli P	2017	2012–2015	Rome	ET	18	0(0)	0(0)	0(0)	ND
33	Souder E	2017	2007–2014	USA	AD	30	0(0)	0(0)	ND	ND
34	Lönnqvist E	2018	2006–2017	Finland	ET	50	0(0)	0(0)	ND	ND
35	Li LJ	2019	2013–2018	China	ET	32	25(78.1)	25(78.1)	25(78.1)	25(78.1)
36	Li L	2019	2014–2016	China	ET	335	292(87.2)	292(87.2)	292(87.2)	292(87.2)
37	Zhang JS	2019	2015–2017	China	ET	105	51(48.6)	51(48.6)	51(48.6)	51(48.6)
38	Hua CZ	2019	2016	China	ET	126	95(75.4)	95(75.4)	ND	95(75.4)
39	Fu P	2019	2016–2017	China	ET	141	81(57.4)*	81(57.4)	81(57.4)*	ND
40	Xu Z	2019	2012–2015	China	ET	167	ND	163(97.6)	ND	ND
41	Wu DX	2019	2016–2017	China	ET	36	26(72.2)	26(72.2)	ND	ND
42	Zhang Z	2021	2017	China	ET	211	138(65.4)	138(65.4)	ND	138(65.4)
43	Lin X	2021	2017–2018	China	ET	55	ND	25(45.5)	ND	25(45.5)
44	Ma F-y	2021	2016–2018	China	ET	86	49(57.0)	49(57.0)	ND	49(57.0)
45	Zaytsev EM	2021	2001–2010	Russia	BM	7	ND	1(14.3)	ND	ND
46	Mi YM	2021	2016–2018	China	ET	125	78(62.4)	78(62.4)	ND	ND
47	Lin L	2022	2018–2022	China	ET	130	98(75.4)	98(75.4)	ND	98(75.4)
48	Wu X	2022	2017–2019	China	ET	311	0(0)	265(85.2)	0(0)	0(0)
49	Zhang J	2022	2018–2019	China	ET	60	46(76.7)	46(76.7)	45(75.0)	ND
50	Cai J	2023	2018–2022	China	ET	103	ND	81(78.6)	ND	ND
51	Fu P	2023	2021–2022	China	ET	150	134(89.3)	134(89.3)	134(89.3)	ND
52	He B	2024	2019–2020	China	ET	62	55(88.7)	56(90.3)*	ND	56(90.3)*
53	Guo M	2024	2019–2022	China	ET	192	188(97.9)	ND	ND	ND
54	Wang L	2024	2022	China	ET	30	29(96.7)	ND	ND	ND
55	Marimón JM	2024	2023	Spain	ET	19	ND	0(0)	ND	ND
56	Miettinen M	2024	2024	Finland	ET	462	1(0.2)	1(0.2)	1(0.2)	ND
57	Rodrigues C	2024	2023–2024	France	ET	67	1(1.5)	1(1.5)	1(1.5)	ND

*Note*: For studies involving resistant strains, the breakpoint was set at 256 mg/L (unless specially noted).

*, breakpoint was 32 mg/L; **, breakpoint was 128 mg/L.

BM, broth dilution method; ND, not determined; AD, agar dilution; ET, Epsilometer test. Resistant No (%): number of resistant isolates (number of resistant isolates/number of isolates*100%).

Based on the integrated data regarding *B. pertussis* drug resistance, the PRR for four antimicrobial agents are summarized in Table 2. Subgroup analyses based on year, country, and AST method are also provided. Forest plots illustrating resistance to each antimicrobial agent and subgroup comparisons are available in Supplementary Table 2.

**Table 2.** Pooled resistance rates for azithromycin, erythromycin, clarithromycin, and clindamycin

Category	Number of isolates	Resistant no. (%)	PRR#	95% CI	Heterogeneity I2 (%)
Erythromycin	5,337	1,839(34.5)	0.21	0.11–0.32	99.07
Subgroup by year					
Before 2010	2,088	20(1.0)	0.00	0–0.01	66.27
2011–2024	3,249	1,819(56.0)	0.42	0.26–0.58	98.86
Subgroup by country					
China	2,410	1,813(75.2)	0.71	0.61–0.81	94.71
Other country	2,927	26(0.9)	0.00	0–0.01	58.33
Subgroup by test					
AD	856	16(1.9)	0.01	0–0.03	72.53
ET	4,474	1,822(40.7)	0.33	0.19–0.49	99.25
Azithromycin	4,707	1,502(31.9)	0.25	0.12–0.40	99.22
Subgroup by year					
Before 2010	1,601	5(0.3)	0.00	0.00–0.00	0
2011–2024	3,106	1,497(48.2)	0.45	0.31–0.59	99.82
Subgroup by country					
China	1,996	1,495(74.9)	0.72	0.61–0.82	98.87
Other country	2,711	7(0.3)	0.00	0–0.00	0
Subgroup by test					
AD	472	0(0.0)	0.00	0–0.00	0
ET	4,235	1,502(35.5)	0.37	0.20–0.55	99.35
Clarithromycin	3,144	735(23.4)	0.15	0.04–0.30	99.11
Subgroup by year					
Before 2010	1,407	6(0.4)	0.00	0–0.01	0
2011–2024	1,737	729(42.0)	0.35	0.18–0.53	99.63
Subgroup by country					
China	982	724(73.7)	0.64	0.41–0.84	96.18
Other country	2,162	11(0.5)	0.00	0.00–0.00	37.54
Subgroup by test					
AD	340	3(0.9)	0.00	0–0.00	47.47
ET	2,804	732(26.1)	0.22	0.07–0.43	99.32
Clindamycin	1,443	926(64.2)	0.49	0.28–0.70	97.9
Subgroup by year (country)					
Before 2010 (other country)	170	1(0.6)	0.00	0–0.01	28.87
2011–2024 (China)	1,273	925(72.7)	0.71	0.61–0.80	91.41

*Note*: The resistance breakpoint was set at 32 mg/L for all four antibiotics.

#PRR, pooled resistance rate; CI, confidence interval; Resistant No. (%): number of resistant isolates (number of resistant isolates/number of isolates*100%).

#### Resistance to erythromycin

A total of 53 studies involving 5,337 *B. pertussis* isolates assessed erythromycin susceptibility. Among these, 1,839 isolates (34.5%) were reported as resistant. The PRR was 21% (95% CI 11–32%) with substantial heterogeneity (*I^2^* = 99.07%), and Egger’s test indicated no publication bias (*p* = 0.95).

The subgroup analysis that compared the data from China (PRR 71%; 95% CI 61–81%) and other countries (PRR 0%; 95% CI 0–1%) indicated that macrolide-resistant *B. pertussis* strains are highly prevalent in China but rarely observed in other regions. This difference was statistically significant (*p* < 0.01). The groups differed in AST (*p* < 0.01), with higher rates in ET (33%, 95% CI 19–49%) and lower in AD (1%, 95% CI 0–3%). By analysing the data of 1994–2010 (PRR 0%; 95% CI 0–1%) and 2011–2024 (PRR 42%; 95% CI 26–58%), we found a significant increase in the resistance rate (*p* < 0.01).

#### Resistance to azithromycin

Susceptibility to azithromycin was determined in 40 studies where 4,707 *B. pertussis* isolates were tested. According to the statistics, 1,502 (31.9%) of tested isolates were considered resistant, and the PRR was 25% (95% CI 12–40%) with substantial heterogeneity (*I^2^* = 99.22%). Egger’s test indicated no publication bias (*p* = 0.33).

The subgroup analysis based on the AST studies showed that the resistance rates differ between the groups (*p* < 0.01); the PRR was 37% (95% CI 20–55%) by ET and 0 (95% CI 0–0%) by AD. Interestingly, almost all studies from China reported azithromycin-resistant isolates, except one study [18]. The raw resistance rate was analysed, showing China with a PRR at 72% (95% CI 61–82%) and other countries with a PRR 0% (95% CI 0–0%). This difference was obviously statistically significant (*p* < 0.01). By analysing the data of 1994–2010 (PRR 0%; 95% CI 0–0%) and 2011–2024 (PRR 45%; 95% CI 31–59%), we found a significant increase in the resistance rate (*p* < 0.01).

#### Resistance to clarithromycin

Susceptibility of *B. pertussis* to clarithromycin was evaluated across 26 studies, encompassing a total of 3,144 isolates. Among these, 735 isolates (23.4%) exhibited resistance. The overall PRR was 15% (95% CI 4–30%), with a substantial heterogeneity (*I^2^* = 99.11%). Egger’s test indicated no publication bias (*p* = 0.73).

The subgroup analysis found a significant difference (*p* < 0.01) between China (PRR 64%; 95% CI 41–84%) and other countries (PRR 0%; 95% CI 0–0%). Based on the AST of studies, the resistance rates also differed between groups (*p* < 0.01), exhibiting a higher PRR in the ET group (22%; 95% CI 7–43%) and a very low PRR in the AD group (0%; 95% CI 0–0%). A subgroup analysis, a comparison of 1994–2010 and 2011–2024 data, showed a significant difference (*p* < 0.01), 0% (95% CI 0–1%) *versus* 35% (95% CI 18–53%).

#### Resistance to clindamycin

Meanwhile, susceptibility to clindamycin was investigated in 15 studies. A total of 1,443 *B. pertussis* isolates were surveyed, and 926 isolates were reported as resistant (64.2%); the total PRR was 49% (95%CI 28–70%), with high heterogeneity (*I^2^* = 97.9%). Egger’s test indicated publication bias (*p* = 0.04), which means long-term data monitoring was necessary. All studies employed the ET method. Interestingly, all the studies conducted in China were reported during 2011–2024 (PRR 71%; 95% CI 61–80%). Meanwhile, research from other countries was published between 1994 and 2010 (PRR 0%; 95% CI 0–1%).

#### Resistance to quinolone

As we know, there was only one study that reported the resistance to quinolone. Six *B. pertussis* strains isolated from 2004 to 2006 in Japan showed high-level resistance to nalidixic acid (NAL; MIC >256 mg/L).

### *MIC*_***90***_ ***of B. pertussis***

MIC_90_ values were collected from all 77 included studies [18–94]. Among these, 40 studies reported the MIC_90_ of azithromycin. With the exception of 18 studies that indicated an MIC_90_ of 256 mg/L, the remaining studies reported MIC_90_s ranging from 0.013 mg/L to 0.19 mg/L. For erythromycin, 54 studies provided MIC_90_ data; 17 of these reported an MIC_90_ of 256 mg/L, while the majority indicated a range of 0.013 mg/L to 4 mg/L. Twenty-four studies reported MIC_90_ values for clarithromycin, which ranged from 0.013 mg/L to 8 mg/L, and for clindamycin, which ranged from 0.19 mg/L to 2 mg/L, excluding reports involving resistant strains. The MIC_90_ values of several common antimicrobial agents – including penicillins, cephalosporins, quinolones, tetracyclines, aminoglycosides, and carbapenems – against *B. pertussis* are summarized in Table 3. The number of studies and the number of isolates for each antibiotic are also included.

**Table 3.** Reported ranges of the minimum inhibitory concentration required to inhibit 90% of isolates (MIC_90_) of common antimicrobial agents against *Bordetella pertussis*

Penicillins	MIC_90_(mg/L)	No. of studies (Isolates)	Cephalosporins	MIC_90_ (mg/L)	No. of studies (Isolates)
Amoxicillin	0.064–10	13(979)	Cephradine	128	1(28)
Amoxicillin-clavulanate	0.5–2	2(66)	Cefadroxil	128	1(28)
Ampicillin	0.09–2	17(1,643)	Cefalexin	50.6–128	6(426)
Ampicillin-sulbactam	0.19	1(126)	Cefazolin	10.9–12.5	2(84)
Piperacillin	0.006–0.156	8(673)	Cefaclor	20–100	5(289)
Piperacillin-tazobactam	<0.016	2(673)	Cefprozil	64	1(33)
**Macrolides**	**MIC**_**90**_**(mg/L)**	**No. of studies (isolates)**	Cefuroxime	5.3–16	5(788)
Roxithromycin*	0.03–0.5	8(809)	Cefotetan	2	1(28)
Erythromycin*	0.013–4	54(4,624)	Ceftriaxone	0.04–2	11(1,074)
Azithromycin*	0.013–0.19	40(3,981)	Ceftazidime	0.125–0.78	7(801)
Clarithromycin*	0.013–8	24(2,330)	Cefotaxime	0.064–0.625	6(404)
**Quinolones**	**MIC**_**90**_**(mg/L)**	**No. of studies (isolates)**	Cefoperazone	0.006–0.078	5(336)
Nalidixic acid*	1.25–6.25	5(187)	Cefoperazone-sulbactam	0.047–0.094	4(548)
Ciprofloxacin	0.01–2	18(1,151)	Cefixime	1.25–16	2(60)
Levofloxacin	0.01–1	11(767)	Cefdinir	32–128	3(120)
Moxifloxacin	0.012–0.03	2(134)	Cefpodoxime	16–100	4(129)
Norfloxacin	0.094–1	5(160)	Ceftibuten	128	1(33)
Gemifloxacin	0.03	1(102)	Cefetamet	64–100	2(71)
**Tetracyclines**	**MIC**_**90**_**(mg/L)**	**No. of studies (isolates)**	Cefpirome	1	1(34)
Tetracycline	0.2–32	9(1,008)	Cefepime	0.125	1(16)
Doxycycline	0.12–6	4(547)	**Miscellaneous agents**	**MIC**_**90**_**(mg/L)**	**No. of studies (isolates)**
Tigecycline	0.016	1(100)	Chloramphenicol	0.4–1	4(185)
Minocycline	0.03–16	6(325)	Spectinomycin	1.25–1.4	1(100)
**Aminoglycosides**	**MIC**_**90**_**(mg/L)**	**No. of studies (isolates)**	Rifampin	0.125–16	8(742)
Amikacin	8	2(367)	Polymyxin B	0.076–0.099	1(100)
Gentamicin	1	2(367)	Trimethoprim-sulfamethoxazole	0.01–0.75	28(2,790)
Tobramycin	1	2(367)	Aztreonam	0.5–4	2(44)
Streptomycin	2.5–3.1	2(117)	Latamoxef	0.078–0.147	3(252)
**Carbapenems**	**MIC**_**90**_**(mg/L)**	**No. of studies (isolates)**	Cefoxitin	2.1–4	3(112)
Meropenem	0.023–2	11(1,119)	Cefmetazole	2.5	1(27)
Imipenem	0.05–32	5(494)	Clindamycin*	0.19–2	13(1,305)

* In addition to the MIC_90_ ranges listed, values as high as 256 mg/L were reported in some studies due to a high prevalence of resistant isolates.

## Discussion

The primary objective of this meta-analysis was to evaluate macrolide resistance rates in *B. pertussis* to assess the current therapeutic utility of this drug class. Due to its shared mechanism of action and significant cross-resistance with macrolides, clindamycin was also included in the analysis. To enhance the reliability of resistance data, case reports and studies involving fewer than five isolates were excluded, as smaller sample sizes are prone to increased bias in resistance rate estimation [16]. Due to the fastidious growth requirements of *B. pertussis*, AST is performed only in a limited number of tertiary hospitals, primarily for research purposes rather than routine diagnosis [58, 63]. During the actual statistical assessment, it was observed that the number of studies reporting resistant strains was limited, with only 57 studies meeting the inclusion criteria. Among these, a considerable proportion reported a resistance rate of 0%. Therefore, this meta-analysis includes high heterogeneity to some extent. The inclusion of negative results suggests a low risk of publication bias, a conclusion further supported by the results of Egger’s test (*p* > 0.05 in 3 out of 4 analyses).

Consequently, the macrolide resistance rate of *B. pertussis* in China was considerably higher than that reported in other countries, a difference that may be attributable to the extensive use of macrolides in China. Furthermore, macrolide-resistant *B. pertussis* has also been documented in several other countries, including the United States, Russia [80], Iran, France [95], Japan [96], Vietnam [97], and Finland. The true prevalence of macrolide-resistant *B. pertussis* is likely underestimated, largely due to limitations in diagnostic practices. In China, bacterial culture remains a primary diagnostic method. Although culture is time-consuming and may yield false-negative results, it provides viable isolates essential for AST. In contrast, most other countries rely on polymerase chain reaction (PCR) or serology as gold-standard diagnostic techniques due to their high sensitivity and rapid turnaround time [98]. However, a significant drawback of these methods is their inability to provide antibiotic susceptibility profiles, greatly increasing the risk of undetected resistant strains. It is therefore reasonable to suggest – at least theoretically – that high macrolide resistance rates in *B. pertussis* may be overlooked outside of China due to these diagnostic limitations.

Overall, our results indicate that ET was the most commonly used method for AST, followed by AD. The AD method may be better suited for testing large numbers of isolates, given that fresh plates must be prepared for each experiment. In contrast, commercially available ET strips allow for individual testing of each isolate. It is worth noting that China, which accounts for the majority of reported macrolide-resistant *B. pertussis* strains, demonstrates a clear preference for the use of ET over AD in research settings. This preference may explain the statistically significant differences observed between the AD and ET groups in the subgroup analysis. The earliest study included in this review was published in 1967. However, the first documented case of erythromycin-resistant *B. pertussis* was reported in Arizona in 1994 [14], while macrolide-resistant strains were not identified in China until 2013 [99]. To better understand this temporal trend, we performed a subgroup analysis by categorizing publication dates into periods before and after 2010. The results clearly indicate a marked increase in macrolide-resistant *B. pertussis* strains reported within the past decade.

In 2003, Bartkus and colleagues [19] identified the A2047G mutation in the 23S rRNA gene as a mechanism conferring erythromycin resistance in *B. pertussis.* Since then, the vast majority of erythromycin-resistant *B. pertussis* strains have been reported to carry either the A2047G or A2058G point mutation [34, 62–[64, 80, 97, 99]–104], with only a limited number of isolates lacking these mutations [34, 69]. However, the presence of a small number of isolates lacking these mutations suggests that additional, yet unidentified, resistance mechanisms may also exist. This phenomenon indicates that although homologous resistance is currently predominant in *B. pertussis*, continuous monitoring for the emergence of heteroresistance remains crucial, and *in vitro* AST of *B. pertussis* strains is still necessary. In 2009, Ohtsuka and colleagues [72] reported quinolone resistance in *B. pertussis.* Six clinical isolates from Japan exhibited high-level resistance to nalidixic acid (NAL; MIC>256 mg/L), which was attributed to a mutation in the quinolone resistance-determining region (QRDR) of the gyrA gene. To the best of our knowledge, no other quinolone-resistant strains have been reported elsewhere. Nevertheless, continued surveillance is essential to monitor for the emergence of additional mutations that may confer further antibiotic resistance.

The CLSI and the EUCAST have not yet established official breakpoints for AST of *B. pertussis.* Therefore, to evaluate the exposure of *B. pertussis* to various antibiotics, we have summarized the MIC_90_ values of commonly tested antibiotics reported in the 77 included studies. This compilation provides a valuable reference for future research.

Since *B. pertussis* is difficult to culture and testing for the bacteria by any strategy must be specifically requested, treatment for serious respiratory illness is empirical. Macrolide antibiotics, particularly azithromycin, are frequently endorsed for respiratory tract infections and behave actively against *B. pertussis*, according to previous experiments in the 20th century. Azithromycin remains one of the recommended drugs for the treatment and prophylaxis of pertussis, yet a substitute medication is required, especially in China, due to the high resistance rate. In fact, macrolide-resistant isolates have been shown to have a worse clearance rate at 7 and 14 days post-treatment [68]. For paediatric patients who have failed macrolide therapy, some studies recommend oral sulfamethoxazole (STX) [45, 63]. STX, a second-line agent for pertussis treatment, demonstrated low MIC_90_ values in this review. No resistant *B. pertussis* isolates have been reported to date. However, STX is not recommended for neonates or infants under 2 months of age.

In addition, penicillins and cephalosporins are good alternatives for children. However, it should be noted that *B. pertussis*, as a Gram-negative bacterium, exhibits inherently low susceptibility to first- and second-generation cephalosporins. Actually, cephalexin was used as the inhibitory agent in charcoal horse blood agar. The data on the *in vitro* activities of the new oral cephalosporins must be interpreted as comparable to the concentrations achievable in respiratory secretions. As shown in Table 3, cephalosporins, including cefixime, cefdinir, cefpodoxime, ceftibuten, and cefetamet, all exhibited high MIC values against *B. pertussis*, suggesting that oral cephalosporins may have limited efficacy in the treatment of pertussis. Based on the breakpoints specified by the 35th edition of the CLSI for *H. influenzae*, the MIC_90_ values of penicillins (including amoxicillin-clavulanate, ampicillin-sulbactam, piperacillin, and piperacillin-tazobactam) and intravenous third-generation cephalosporins (such as ceftriaxone, ceftazidime, and cefotaxime) described in Table 3 suggest potential susceptibility of *B. pertussis* to these agents.

In recent years, the incidence of pertussis in adults has also become a notable concern. Therefore, it is necessary to investigate the susceptibility of *B. pertussis* to a broader range of antibiotics in order to guide clinical treatment. Quinolones are currently recommended for the empirical treatment of respiratory tract infections in adult patients [28]. Multiple studies have indicated that certain quinolones exhibit low MIC values against *B. pertussis*, and their concentrations in respiratory secretions are sufficient to exceed the MIC values for the pathogen [25]. Nakamori and colleagues reported that the sputum concentration of levofloxacin reaches 1.27–4.36 mg/L [105], suggesting its potential *in vivo* efficacy against pertussis. Theoretically, quinolones with enhanced antibacterial activity may play an important role in the treatment of pertussis in adults.

The treatment of pertussis in adults appears to be relatively straightforward, as indicated by the low MIC values of numerous antimicrobial agents shown in Table 3. Given that pertussis infection in adults is generally mild, quinolones, SXT, and certain β-lactam antibiotics are considered sufficient for treatment. Other agents were not analysed in this study.

## Conclusion

In summary, there has been a significant increase in macrolide-resistant *B. pertussis* in recent years. China has been particularly affected by this trend, underscoring the need to reevaluate the clinical utility of macrolides in this region and implement enhanced antimicrobial resistance surveillance. In contrast, macrolide resistance remains rare in *B. pertussis* isolates from other countries; however, this may reflect underreporting rather than true susceptibility, emphasizing the continued importance of routine monitoring globally. To date, only six *B. pertussis* strains exhibiting high-level resistance to nalidixic acid have been documented. Nevertheless, ongoing vigilance is essential to detect emerging mutations that may confer additional drug resistance.

Additionally, based on the aggregated data, STX, penicillins (including amoxicillin-clavulanate, ampicillin-sulbactam, piperacillin, and piperacillin-tazobactam), and intravenous third-generation cephalosporins (such as ceftriaxone, ceftazidime, cefotaxime, and cefoperazone-sulbactam) demonstrate strong *in vitro* activity against *B. pertussis*, suggesting their potential as alternative treatments for pertussis in children. For adults, quinolones currently remain effective.

## Supporting information

10.1017/S0950268826101010.sm001 Ma et al. supplementary material 1 Ma et al. supplementary material

10.1017/S0950268826101010.sm002 Ma et al. supplementary material 2 Ma et al. supplementary material

## Data Availability

This systematic review and meta-analysis is based on previously published studies; therefore, no new primary data were generated. All data supporting the findings of this meta-analysis are available within the article and its Supplementary Information files. Specifically: Supplementary Table 1 lists all original studies included in the meta-analysis as well as the quality assessment tables. Supplementary Table 2 contains the forest plots illustrating resistance to each antimicrobial agent and the subgroup comparisons. The study protocol is available on PROSPERO (ID: CRD42022325142). No original code was used beyond the standard functions of the R software (version 4.0.3).

## Supplementary material

The supplementary material for this article can be found at http://doi.org/10.1017/S0950268826101010.
