# Supplementary material for: Antimicrobial resistance in Bordetella pertussis: A systematic review and meta-analysis
Source: Epidemiol Infect. 2026 Feb 6;154:e25. doi: 10.1017/S0950268826101010 (PMC12951334; doi:10.1017/S0950268826101010)
Supplement: Ma et al. supplementary material 1 — Ma et al. supplementary material [file S0950268826101010sup001.docx]

QUALITY ASSESSMENT

| **First Author, Year of Publication** | **Selection (3 points)** | **Comparability (2 points)** | **Outcome (3 points)** | **Total (8 points)** |
| --- | --- | --- | --- | --- |
| Lewis K 1995 [1] | 1 | 1 | 2 | 4 |
| Horikawa K 1995 [2] | 2 | 1 | 1 | 4 |
| Aoyama T 1996 [3] | 1 | 2 | 2 | 5 |
| Cimolai N 1997 [4] | 2 | 1 | 2 | 5 |
| Korgenski EK 1997 [5] | 2 | 1 | 2 | 5 |
| Hoppe JE 1998 [6] | 1 | 2 | 1 | 4 |
| Brett M 1998 [7] | 2 | 2 | 2 | 6 |
| Mortensen JE 2000 [8] | 2 | 1 | 2 | 5 |
| Hill BC 2000 [9] | 2 | 1 | 2 | 5 |
| Gordon KA 2001 [10] | 1 | 2 | 1 | 4 |
| Chodorowska M 2001 [11] | 2 | 2 | 1 | 5 |
| Bartkus JM 2003 [12] | 2 | 2 | 2 | 6 |
| Yamaguchi K 2003 [13] | 1 | 1 | 2 | 4 |
| Bourgeois N 2003 [14] | 1 | 1 | 2 | 4 |
| Ohtsuka M 2004 [15] | 1 | 2 | 1 | 4 |
| Galanakis E 2006 [16] | 2 | 2 | 2 | 6 |
| Sintchenko V 2007 [17] | 2 | 2 | 2 | 6 |
| Yao SM 2008 [18] | 1 | 2 | 1 | 4 |
| Ohtsuka M 2009 [19] | 1 | 1 | 1 | 3 |
| Fry NK 2010 [20] | 2 | 2 | 1 | 5 |
| Luo J 2014 [21] | 1 | 1 | 2 | 4 |
| Horiba K 2014 [22] | 1 | 1 | 2 | 4 |
| Spicer KB 2014 [23] | 1 | 2 | 1 | 4 |
| Shahcheraghi F 2014 [24] | 1 | 2 | 2 | 4 |
| Theofiles AG 2014 [25] | 1 | 1 | 2 | 4 |
| Marchand-Austin A 2014 [26] | 2 | 1 | 2 | 5 |
| Yang Y 2015 [27] | 2 | 2 | 2 | 6 |
| Mirzaei B 2015 [28] | 1 | 1 | 2 | 4 |
| Li Y 2015 [29] | 2 | 2 | 1 | 5 |
| Hardy DJ 2016 [30] | 1 | 2 | 2 | 5 |
| Jakubů V 2017 [31] | 2 | 1 | 2 | 5 |
| Stefanelli P 2017 [32] | 1 | 1 | 2 | 4 |
| Souder E 2017 [33] | 1 | 2 | 1 | 4 |
| Lönnqvist E 2018 [34] | 1 | 2 | 1 | 4 |
| Li LJ 2019 [35] | 1 | 2 | 2 | 5 |
| Li L 2019 [36] | 2 | 2 | 2 | 6 |
| Zhang JS 2019 [37] | 2 | 2 | 2 | 6 |
| Hua CZ 2019 [38] | 2 | 1 | 2 | 5 |
| Fu P 2019 [39] | 2 | 2 | 2 | 6 |
| Xu Z 2019 [40] | 2 | 2 | 2 | 5 |
| Wu DX 2019 [41] | 1 | 2 | 1 | 4 |
| Zhang Z 2021 [42] | 2 | 2 | 2 | 6 |
| Lin X 2021 [43] | 2 | 1 | 2 | 5 |
| Ma F-y 2021 [44] | 2 | 1 | 2 | 5 |
| Zaytsev EM 2021 [45] | 1 | 1 | 2 | 4 |
| Mi YM 2021 [46] | 2 | 1 | 2 | 5 |
| Lin LN 2022 [47] | 2 | 1 | 2 | 5 |
| Wu X 2022 [48] | 2 | 2 | 2 | 6 |
| Zhang J 2022 [49] | 2 | 1 | 2 | 5 |
| Cai J 2023 [50] | 2 | 1 | 1 | 4 |
| Fu P 2023 [51] | 2 | 1 | 2 | 5 |
| He B 2024 [52] | 2 | 2 | 2 | 6 |
| Guo M 2024 [53] | 2 | 2 | 2 | 6 |
| Wang L 2024 [54] | 1 | 2 | 2 | 5 |
| Marimón JM 2024 [55] | 1 | 2 | 2 | 5 |
| Miettinen M 2024 [56] | 2 | 2 | 2 | 6 |
| Rodrigues C 2024 [57] | 2 | 2 | 2 | 6 |

NEWCASTLE - OTTAWA QUALITY ASSESSMENT SCALE

(adapted for cross sectional studies)

Selection: (Maximum 3 points)

1) Representativeness of the sample:

a) Truly representative of the average in the target population. 1 point (all subjects or random sampling)

b) Somewhat representative of the average in the target population. 1 point (non-random sampling)

c) Selected group of users.

d) No description of the sampling strategy.

2) Sample size:

a) Justified and satisfactory. 1 point

b) Not justified.

3) Non-respondents:

a) Comparability between respondents and non-respondents characteristics is established, and the response rate is satisfactory. 1 point

b) The response rate is unsatisfactory, or the comparability between respondents and non-respondents is unsatisfactory.

c) No description of the response rate or the characteristics of the responders and the non-responders.

Comparability: (Maximum 2 points)

1) The subjects in different outcome groups are comparable, based on the study design or analysis. Confounding factors are controlled.

a) The study controls for the most important factor (select one). 1 point

b) The study control for any additional factor. 1 point

Outcome: (Maximum 3 points)

1) Assessment of the outcome:

a) Independent blind assessment. 2 points

b) Record linkage. 2 points

c) Self report. 1 point

d) No description.

2) Statistical test:

a) The statistical test used to analyze the data is clearly described and appropriate, and the measurement of the association is presented, including confidence intervals and the probability level (p value). 1 point

b) The statistical test is not appropriate, not described or incomplete.

This scale is based on an adapted version of the Newcastle-Ottawa Quality Assessment Scale for

cohort studies, refer to “Antimicrobial resistance in Clostridioides (Clostridium) difficile derived from humans: a systematic review and meta-analysis”

In our scale, we have specifically assigned one star for self-reported outcomes, because

our study measures the resistance to antimicrobials, and MIC data require two or more technicians to record.

1. **Lewis K *et al***: Pertussis caused by an erythromycin-resistant strain of Bordetella pertussis. *The Pediatric infectious disease journal* 1995, **14**:388-391.
2. **Horikawa K *et al***: [Prevalence of pertussis in Fukuoka: incidence and MICs of antibiotics for Bordetella pertussis isolate]. *Kansenshogaku zasshi The Journal of the Japanese Association for Infectious Diseases* 1995, **69**:878-883.
3. **Aoyama T *et al***: Efficacy of short-term treatment of pertussis with clarithromycin and azithromycin. *The Journal of pediatrics* 1996, **129**:761-764.
4. **Cimolai N, Zaher A, Trombley C**: Correlation of erythromycin agar dilution susceptibility testing with disc diffusion susceptibility for Bordetella pertussis. *International journal of antimicrobial agents* 1997, **9**:113-116.
5. **Korgenski EK, Daly JA**: Surveillance and detection of erythromycin resistance in Bordetella pertussis isolates recovered from a pediatric population in the Intermountain West region of the United States. *Journal of clinical microbiology* 1997, **35**:2989-2991.
6. **Hoppe JE, Bryskier A**: In vitro susceptibilities of Bordetella pertussis and Bordetella parapertussis to two ketolides (HMR 3004 and HMR 3647), four macrolides (azithromycin, clarithromycin, erythromycin A, and roxithromycin), and two ansamycins (rifampin and rifapentine). *Antimicrobial agents and chemotherapy* 1998, **42**:965-966.
7. **Brett M, Short P, Beatson S**: The comparative in-vitro activity of roxithromycin and other antibiotics against Bordetella pertussis. *The Journal of antimicrobial chemotherapy* 1998, **41 Suppl B**:23-27.
8. **Mortensen JE, Rodgers GL**: In vitro activity of gemifloxacin and other antimicrobial agents against isolates of Bordetella pertussis and Bordetella parapertussis. *The Journal of antimicrobial chemotherapy* 2000, **45 Suppl 1**:47-49.
9. **Hill BC, Baker CN, Tenover FC**: A simplified method for testing Bordetella pertussis for resistance to erythromycin and other antimicrobial agents. *Journal of clinical microbiology* 2000, **38**:1151-1155.
10. **Gordon KA *et al***: Antimicrobial susceptibility testing of clinical isolates of Bordetella pertussis from northern California: report from the SENTRY Antimicrobial Surveillance Program. *Antimicrobial agents and chemotherapy* 2001, **45**:3599-3600.
11. **Chodorowska M, Kuklińska D, Tyski S**: [Susceptibility to macrolide antibiotics of Bordetella pertussis and Bordetella parapertussis strains isolated from whooping cough patients in 1968 and in 1997-99]. *Medycyna doswiadczalna i mikrobiologia* 2001, **53**:39-43.
12. **Bartkus JM *et al***: Identification of a mutation associated with erythromycin resistance in Bordetella pertussis: implications for surveillance of antimicrobial resistance. *Journal of clinical microbiology* 2003, **41**:1167-1172.
13. **Yamaguchi K, Miyazaki S, Okamoto H**: In vitro antibacterial activity and in vivo protective effect of telithromycin - The antibacterial activity against clinical isolates and in vivo protective effect. *Japanese Journal of Chemotherapy* 2003, **51**:55-65.
14. **Bourgeois N, Ghnassia JC, Doucet-Populaire F**: In vitro activity of fluoroquinolones against erythromycin-susceptible and -resistant Bordetella pertussis. *The Journal of antimicrobial chemotherapy* 2003, **51**:742-743.
15. **Ohtsuka M *et al***: [Susceptibility testing and molecular epidemiology of clinical strains of Bordetella pertussis isolated in Japan from 2001 to 2002]. *Kansenshogaku zasshi The Journal of the Japanese Association for Infectious Diseases* 2004, **78**:420-427.
16. **Galanakis E *et al***: Antimicrobial susceptibility of Bordetella pertussis isolates in the state of Washington. *International journal of antimicrobial agents* 2007, **29**:609-611.
17. **Sintchenko V, Brown M, Gilbert GL**: Is Bordetella pertussis susceptibility to erythromycin changing? MIC trends among Australian isolates 1971-2006. *The Journal of antimicrobial chemotherapy* 2007, **60**:1178-1179.
18. **Yao SM *et al***: Antimicrobial susceptibility testing of Bordetella pertussis in Taiwan prompted by a case of pertussis in a paediatric patient. *Journal of medical microbiology* 2008, **57**:1577-1580.
19. **Ohtsuka M *et al***: Emergence of quinolone-resistant Bordetella pertussis in Japan. *Antimicrobial agents and chemotherapy* 2009, **53**:3147-3149.
20. **Fry NK *et al***: Antimicrobial susceptibility testing of historical and recent clinical isolates of Bordetella pertussis in the United Kingdom using the Etest method. *European journal of clinical microbiology & infectious diseases : official publication of the European Society of Clinical Microbiology* 2010, **29**:1183-1185.
21. **Luo J *et al***: [Clinical characteristics of whooping cough in neonates and antimicrobial resistance of the pathogenic bacteria]. *Zhongguo dang dai er ke za zhi = Chinese journal of contemporary pediatrics* 2014, **16**:975-978.
22. **Horiba K *et al***: Clinical manifestations of children with microbiologically confirmed pertussis infection and antimicrobial susceptibility of isolated strains in a regional hospital in Japan, 2008-2012. *Japanese journal of infectious diseases* 2014, **67**:345-348.
23. **Spicer KB *et al***: Occurrence of 3 Bordetella species during an outbreak of cough illness in Ohio: epidemiology, clinical features, laboratory findings and antimicrobial susceptibility. *The Pediatric infectious disease journal* 2014, **33**:e162-167.
24. **Shahcheraghi F *et al***: The First Macrolide-Resistant Bordetella pertussis Strains Isolated From Iranian Patients. *Jundishapur journal of microbiology* 2014, **7**:e10880.
25. **Theofiles AG *et al***: Pertussis Outbreak, Southeastern Minnesota, 2012. *Mayo Clinic proceedings* 2014, **89**:1378-1388.
26. **Marchand-Austin A *et al***: Surveillance of antimicrobial resistance in contemporary clinical isolates of Bordetella pertussis in Ontario, Canada. *International journal of antimicrobial agents* 2014, **44**:82-84.
27. **Yang Y *et al***: Variation in Bordetella pertussis Susceptibility to Erythromycin and Virulence-Related Genotype Changes in China (1970-2014). *PloS one* 2015, **10**:e0138941.
28. **Mirzaei B *et al***: Isolation of High Level Macrolide Resistant Bordetella pertussis Without Transition Mutation at Domain V in Iran. *Jundishapur journal of microbiology* 2015, **8**:e18190.
29. **Li Y *et al***: Where macrolide resistance is prevalent. *APMIS : acta pathologica, microbiologica, et immunologica Scandinavica* 2015, **123**:361-363.
30. **Hardy DJ, Vicino D, Fernandes P**: In Vitro Activity of Solithromycin against Bordetella pertussis, an Emerging Respiratory Pathogen. *Antimicrobial agents and chemotherapy* 2016, **60**:7043-7045.
31. **Jakubů V *et al***: Trends in the Minimum Inhibitory Concentrations of Erythromycin, Clarithromycin, Azithromycin, Ciprofloxacin, and Trimethoprim/Sulfamethoxazole for Strains of Bordetella pertussis isolated in the Czech Republic in 1967-2015. *Central European journal of public health* 2017, **25**:282-286.
32. **Stefanelli P *et al***: Severe pertussis infection in infants less than 6 months of age: Clinical manifestations and molecular characterization. *Human vaccines & immunotherapeutics* 2017, **13**:1073-1077.
33. **Souder E *et al***: Antimicrobial Susceptibility and Molecular Detection of Pertactin-producing and Pertactin-Deficient Bordetella pertussis. *The Pediatric infectious disease journal* 2017, **36**:119-121.
34. **Lönnqvist E *et al***: Antimicrobial susceptibility testing of Finnish Bordetella pertussis isolates collected during 2006-2017. *Journal of global antimicrobial resistance* 2018, **14**:12-16.
35. **Li LJ *et al***: [Antimicrobial susceptibility and antigen genotypes of Bordetella pertussis strains isolated from neonates]. *Zhongguo dang dai er ke za zhi = Chinese journal of contemporary pediatrics* 2019, **21**:208-213.
36. **Li L *et al***: High Prevalence of Macrolide-Resistant Bordetella pertussis and ptxP1 Genotype, Mainland China, 2014-2016. *Expert review of vaccines* 2019, **25**:2205-2214.
37. **Zhang JS *et al***: Clinical characteristics, molecular epidemiology and antimicrobial susceptibility of pertussis among children in southern China. *World journal of pediatrics : WJP* 2020, **16**:185-192.
38. **Hua CZ *et al***: In vitro activity and clinical efficacy of macrolides, cefoperazone-sulbactam and piperacillin/piperacillin-tazobactam against Bordetella pertussis and the clinical manifestations in pertussis patients due to these isolates: A single-centre study in Zhejiang Province, China. *PloS one* 2019, **18**:47-51.
39. **Fu P *et al***: Molecular Evolution and Increasing Macrolide Resistance of Bordetella pertussis, Shanghai, China, 2016-2022. *Emerging infectious diseases* 2023, **30**:29-38.
40. **Xu Z *et al***: Genomic epidemiology of erythromycin-resistant Bordetella pertussis in China. *Emerging microbes & infections* 2019, **8**:461-470.
41. **Wu DX *et al***: Pertussis detection in children with cough of any duration. 2019, **19**:236.
42. **Zhang Z *et al***: Analysis of clinical features,antibiotics-resistance of Bordetella pertussis isolates and treatment outcomes in 211 children with pertussis. *Chinese Journal of Infections Diseases* 2021, **39**:168-174.
43. **Lin X *et al***: Analysis of antibiotic sensitivity and resistance genes of Bordetella pertussis in Chinese children. *Medicine* 2021, **100**:e24090.
44. **Ma F-y *et al***: Drug-resistance of Bordetella pertussis isolated from adults and clinical characteristics of pertussis in adults. *Zhongguo Kangshengsu Zazhi* 2021, **46**:879-883.
45. **Zaytsev EM *et al***: Sensitivity of biofilms of vaccine and freshly isolated Bordetella pertussis strains to antibiotics. *Zhurnal Mikrobiologii, Epidemiologii, i Immunobiologii* 2021, **97**:529-534.
46. **Mi YM *et al***: Effect of Macrolides and β-lactams on Clearance of Bordetella pertussis in the Nasopharynx in Children With Whooping Cough. *The Pediatric infectious disease journal* 2021, **40**:87-90.
47. **Lin LN *et al***: Epidemiological and clinical characteristics of pertussis in children and their close contacts in households: A cross-sectional survey in Zhejiang Province, China. *Frontiers in pediatrics* 2022, **10**.
48. **Wu X *et al***: A Cross-Sectional Study Revealing the Emergence of Erythromycin-Resistant Bordetella pertussis Carrying ptxP3 Alleles in China. *Frontiers in microbiology* 2022, **13**.
49. **Zhang J *et al***: Comparison of macrolide resistance, molecular characteristics and MAST types of Bordetella pertussis collected from Xi'an and Shanghai. *Journal of Xi'an Jiaotong University (Medical Sciences)* 2022, **43**:691-696.
50. **Cai J *et al***: Domination of an emerging erythromycin-resistant ptxP3 Bordetella pertussis clone in Shanghai, China. *International journal of antimicrobial agents* 2023, **62**.
51. **Fu P *et al***: Molecular Evolution and Increasing Macrolide Resistance of Bordetella pertussis, Shanghai, China, 2016-2022. *Emerging infectious diseases* 2023, **30**:29-38.
52. **He B *et al***: Molecular characterization and antimicrobial susceptibility for 62 isolates of Bordetella pertussis from children. *Frontiers in microbiology* 2024, **15**:1498638.
53. **Guo M *et al***: A comparative study on the clinical manifestations of children infected with erythromycin-resistant Bordetella pertussis of ptxPl and ptxP3 genotypes. *Chinese Journal of Applied Clinical Pediatrics* 2024, **39**:89-93.
54. **Wang L *et al***: Analysis of antibiotic resistance and infection of Bordetella pertussis in children with suspected pertussis and close family members. *Chinese Journal of Microbiology and Immunology* 2024, **44**:473-479.
55. **Marimón JM *et al***: Pertussis Outbreak During 2023 in Gipuzkoa, North Spain. *Vaccines* 2024, **12**.
56. **Miettinen M *et al***: Macrolide-resistant Bordetella pertussis strain identified during an ongoing epidemic, Finland, January to October 2024. *Eurosurveillance* 2024, **29.**
57. **Rodrigues C *et al***: Resurgence of Bordetella pertussis, including one macrolide-resistant isolate, France, 2024. *Eurosurveillance* 2024, **29**.
