# Supplementary figures and images for: Antimicrobial resistance in Bordetella pertussis: A systematic review and meta-analysis
Source: Epidemiol Infect. 2026 Feb 6;154:e25. doi: 10.1017/S0950268826101010 (PMC12951334; doi:10.1017/S0950268826101010)

Forrest Plot

**Erythromycin**

**
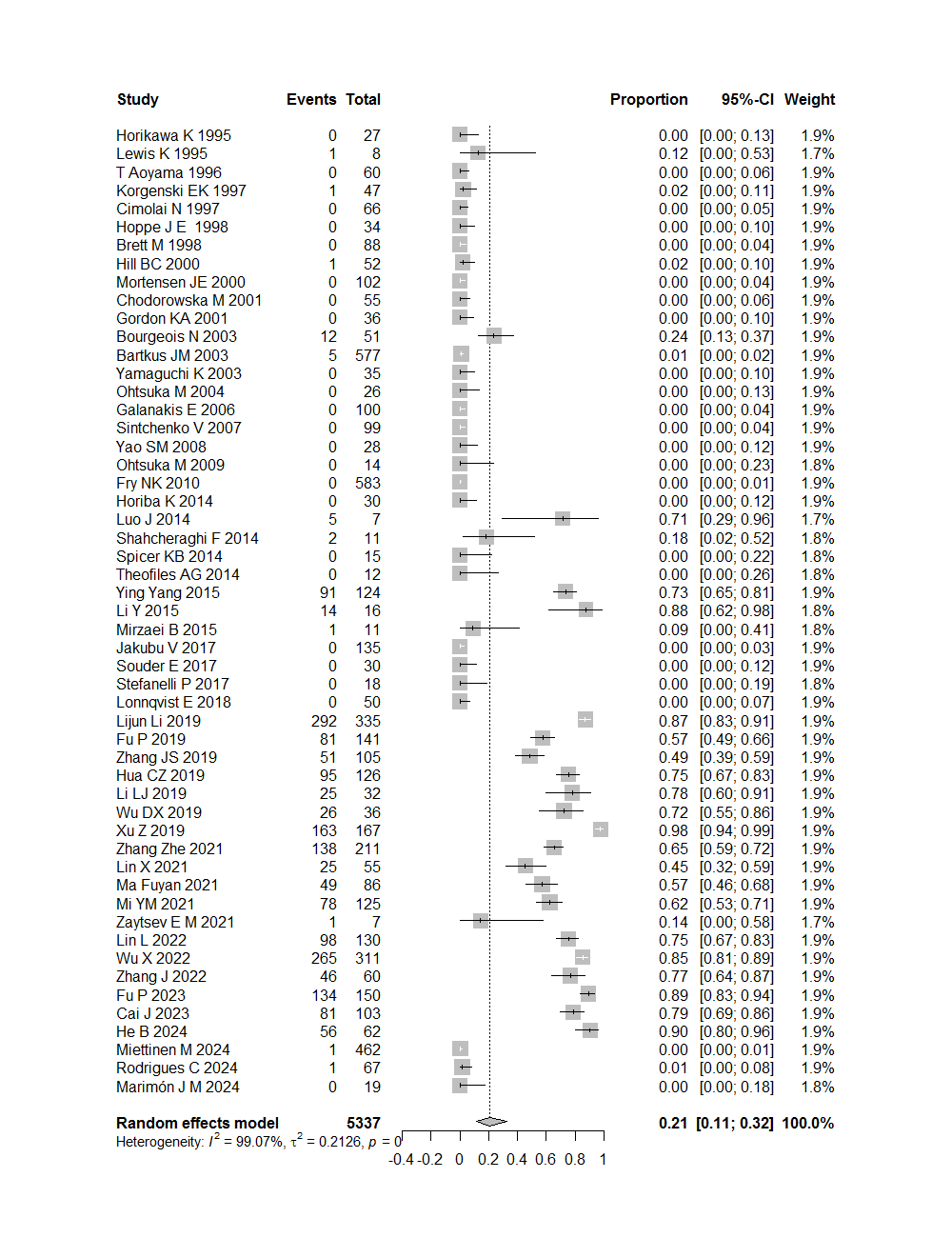
**

**
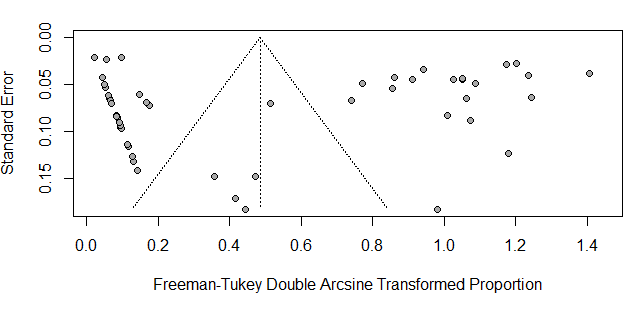
**

**
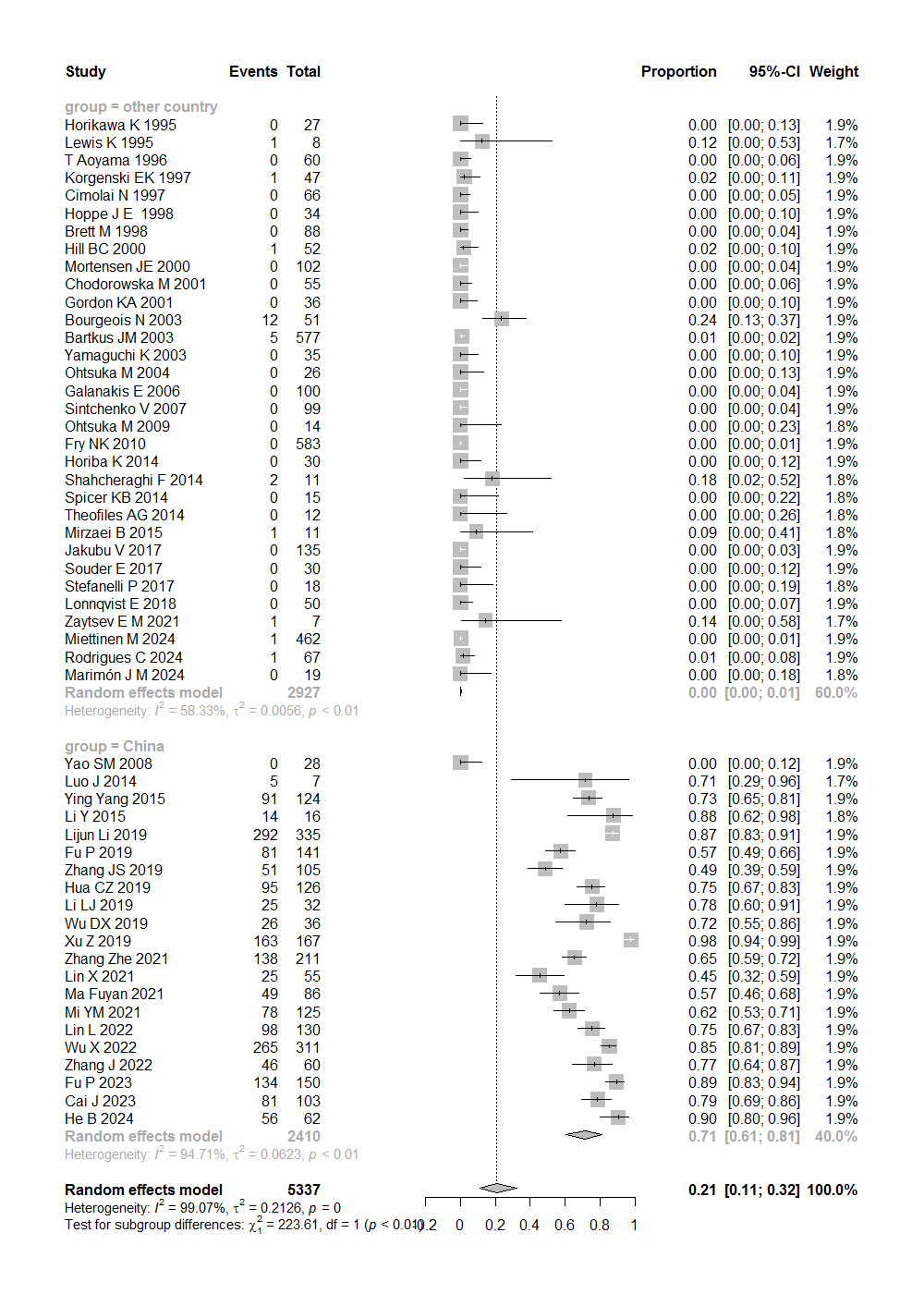
**

**
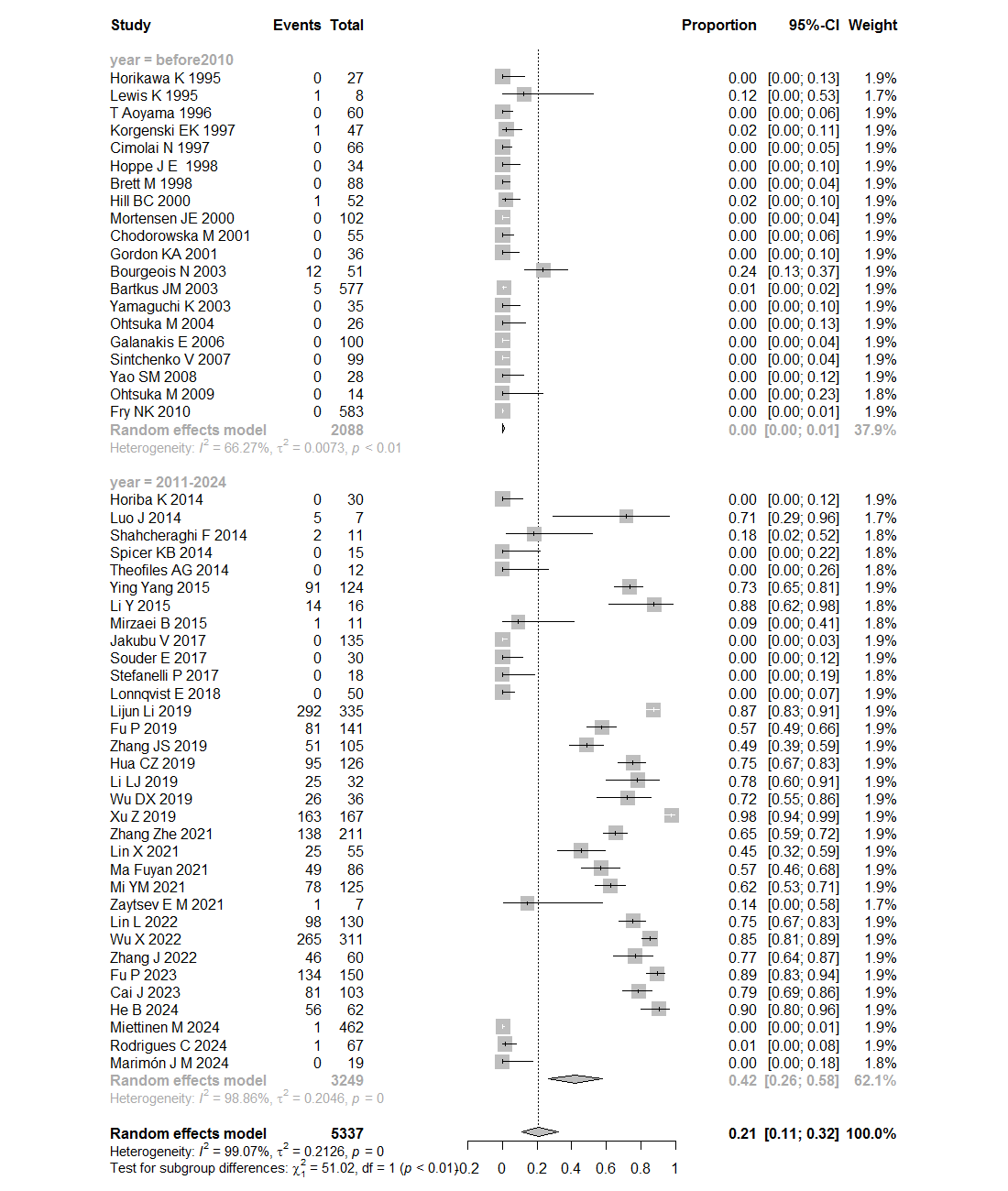
**

**
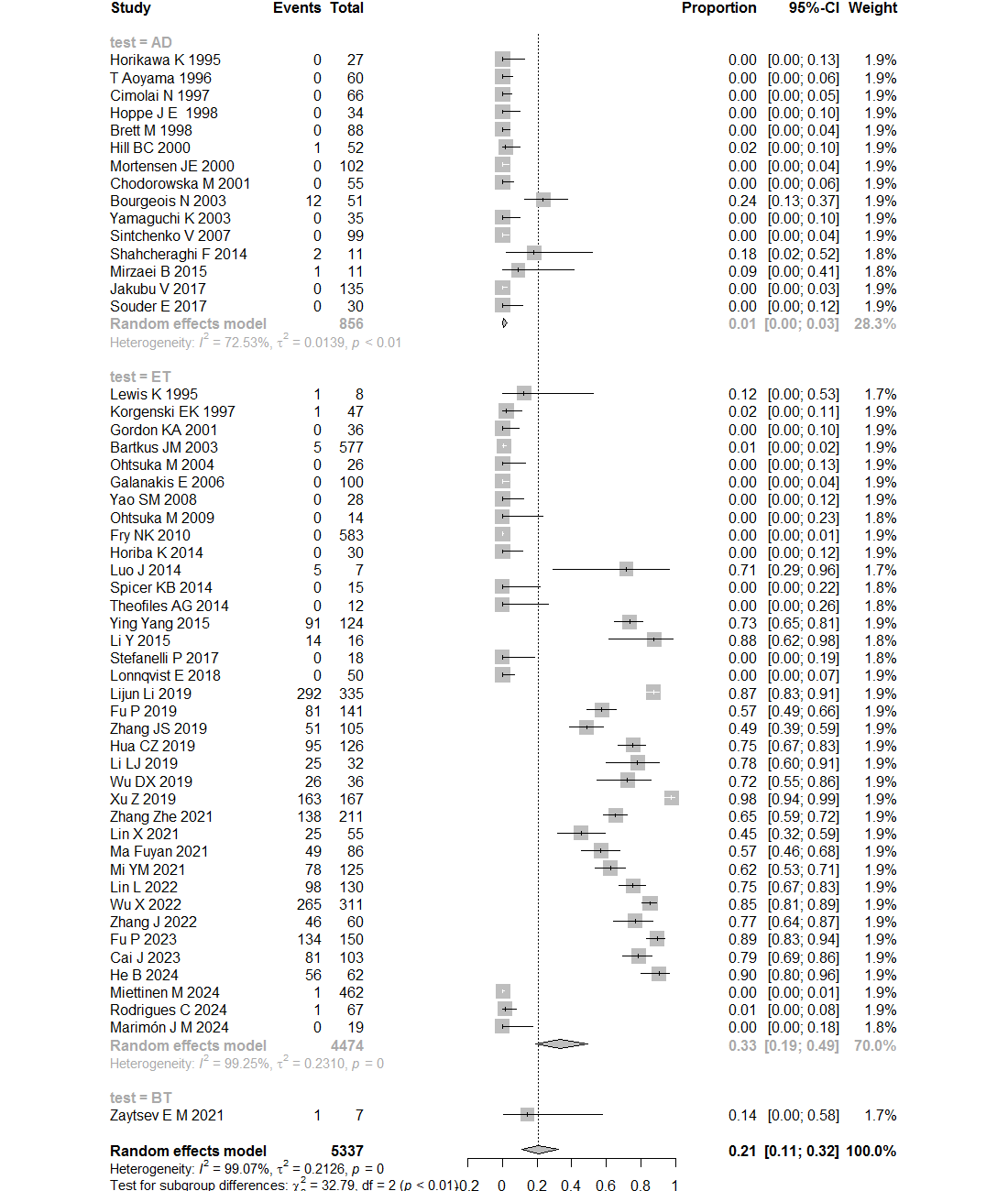
**

**Azithromycin**

**
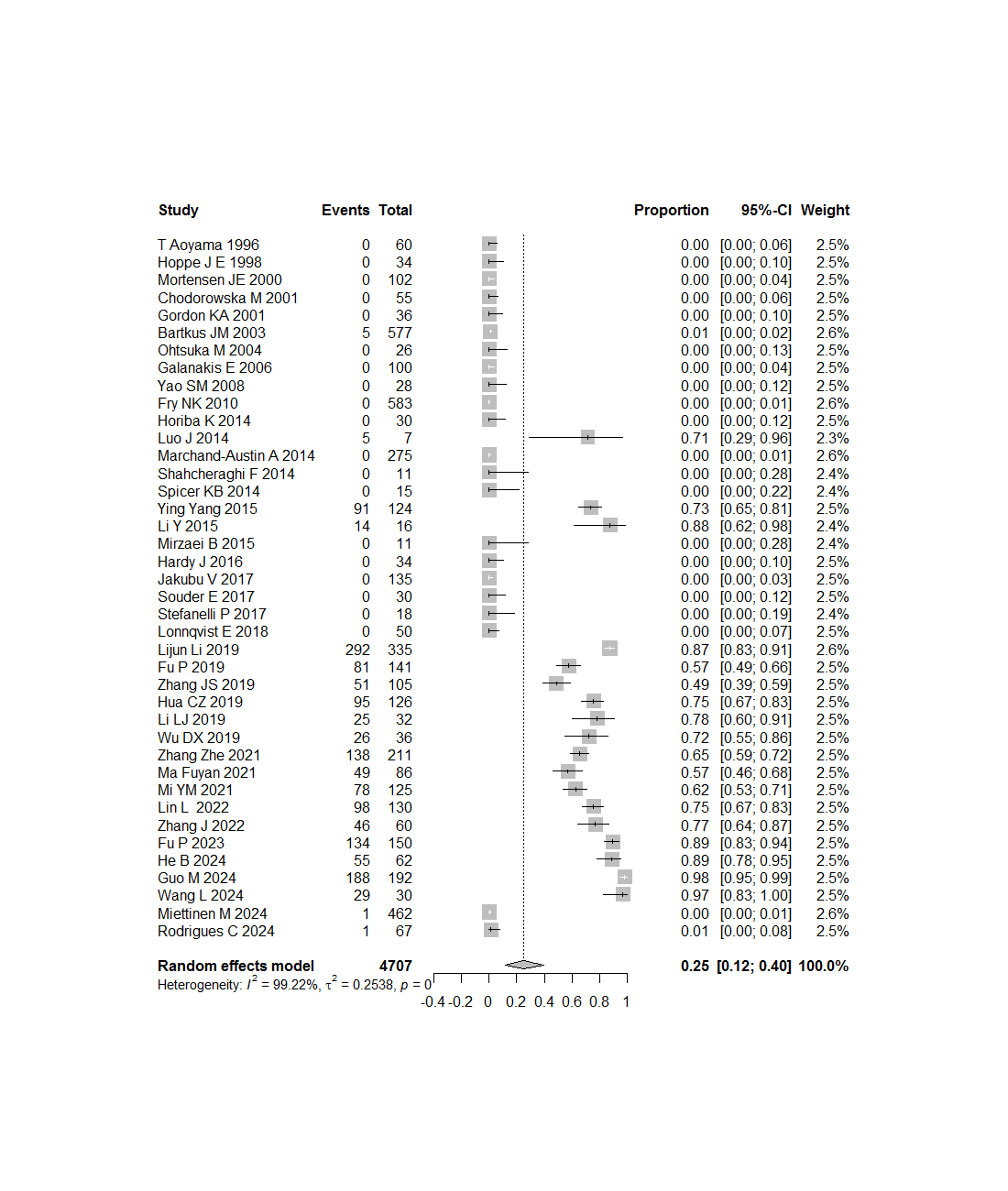
**

**
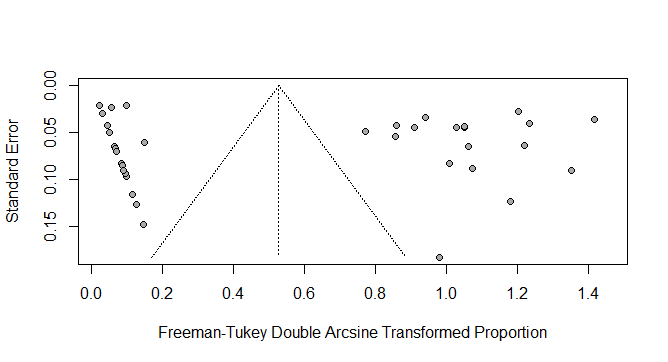
**

**
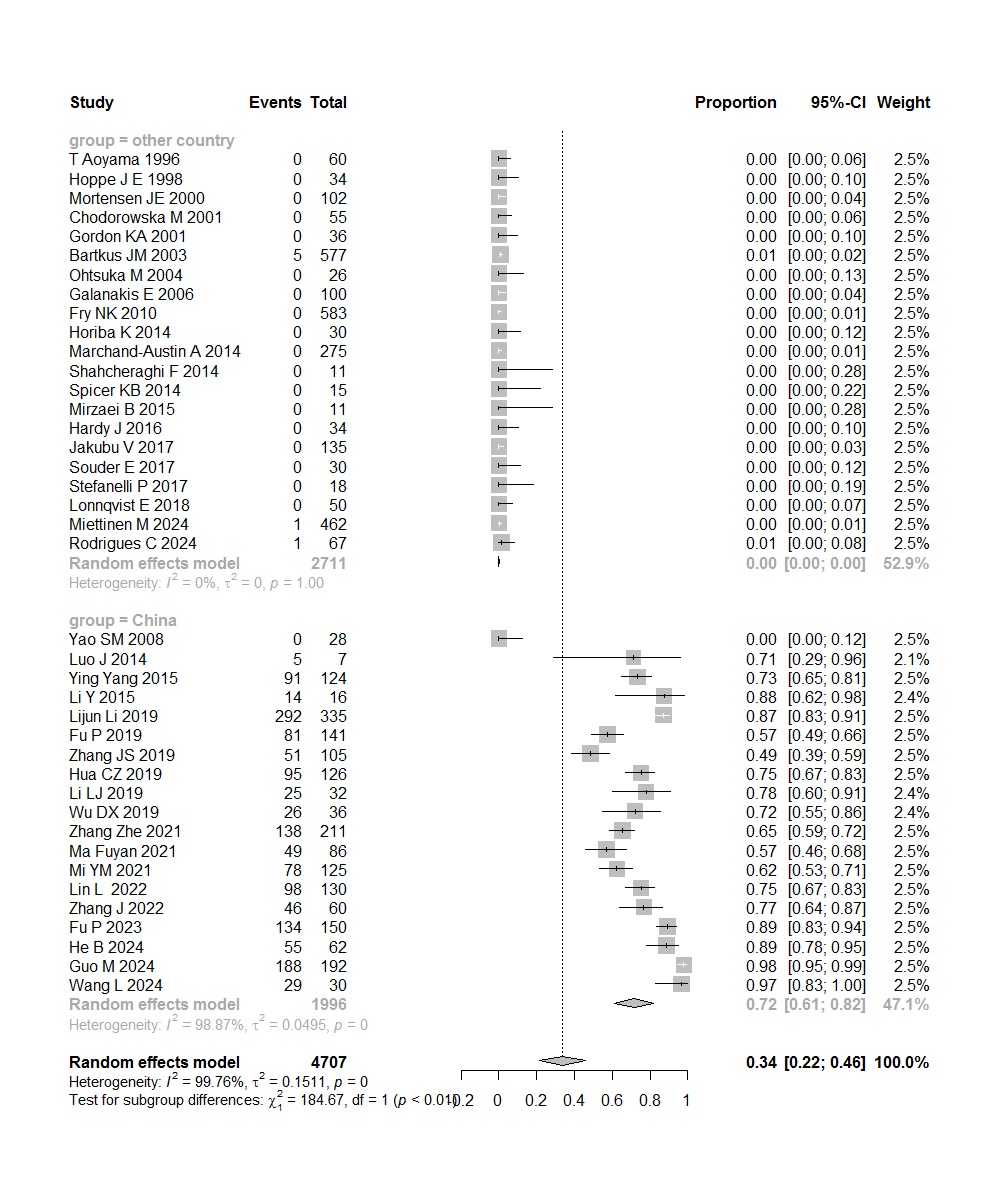
**

**
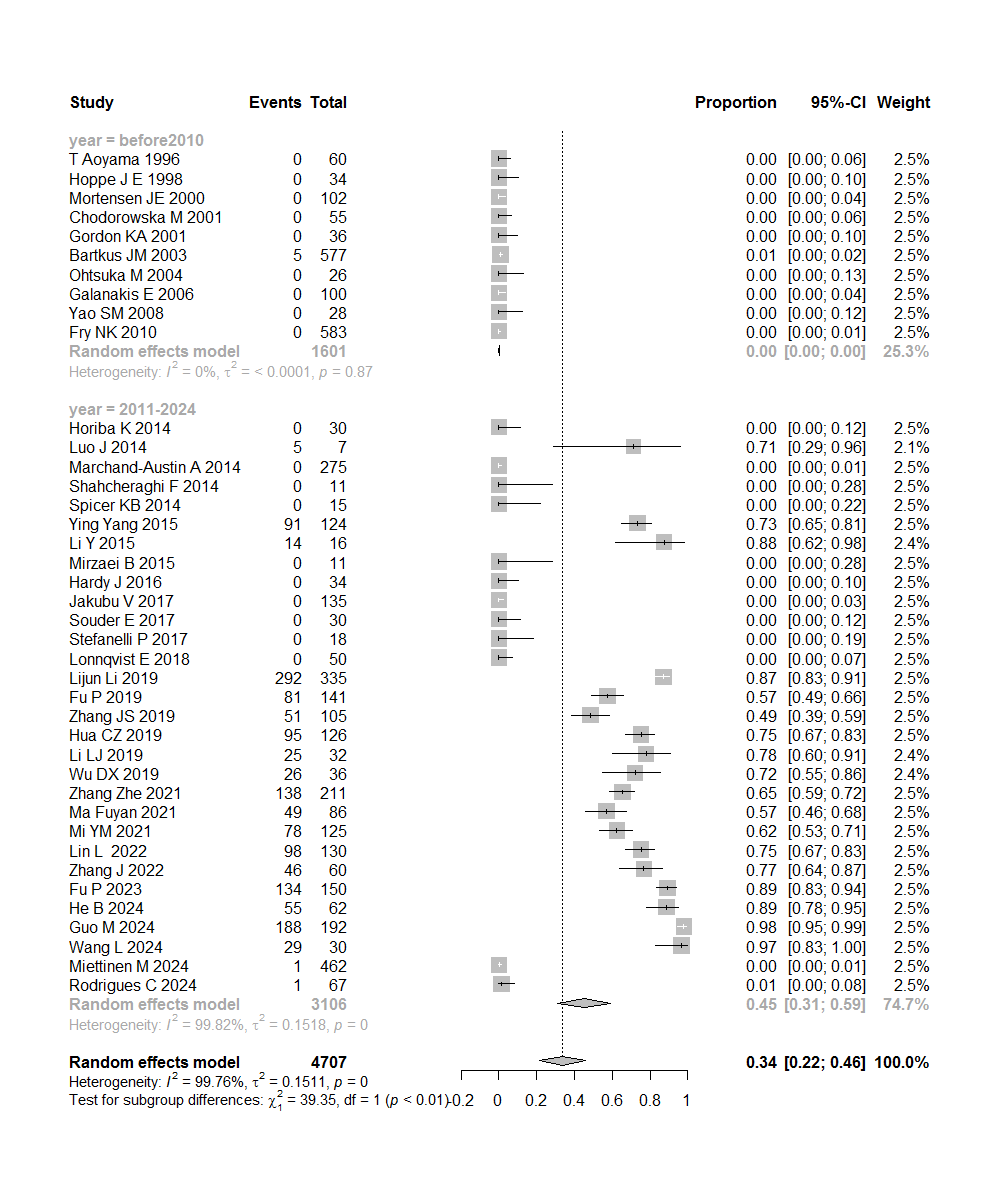
**

**
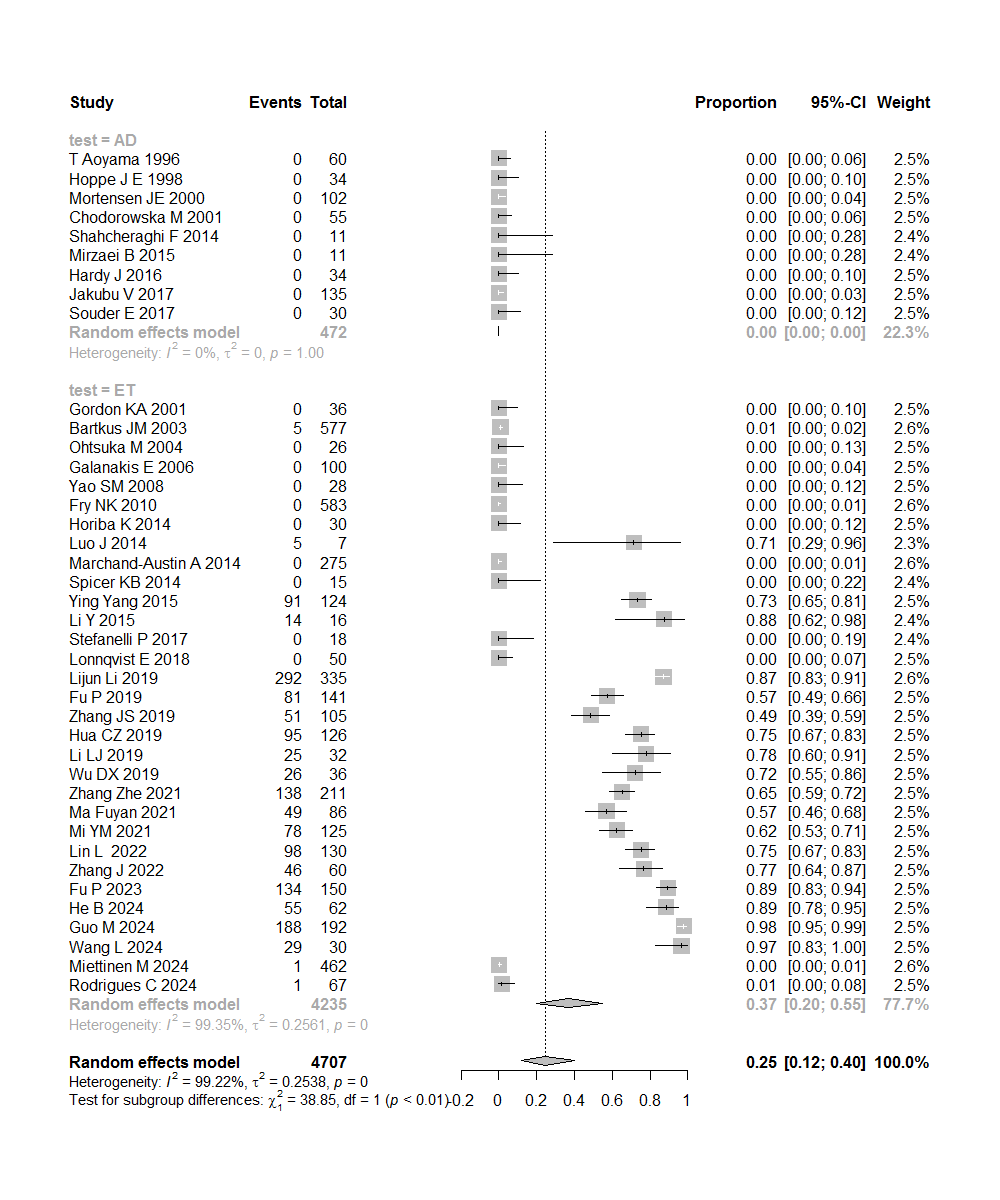
**

**Clarithromycin**


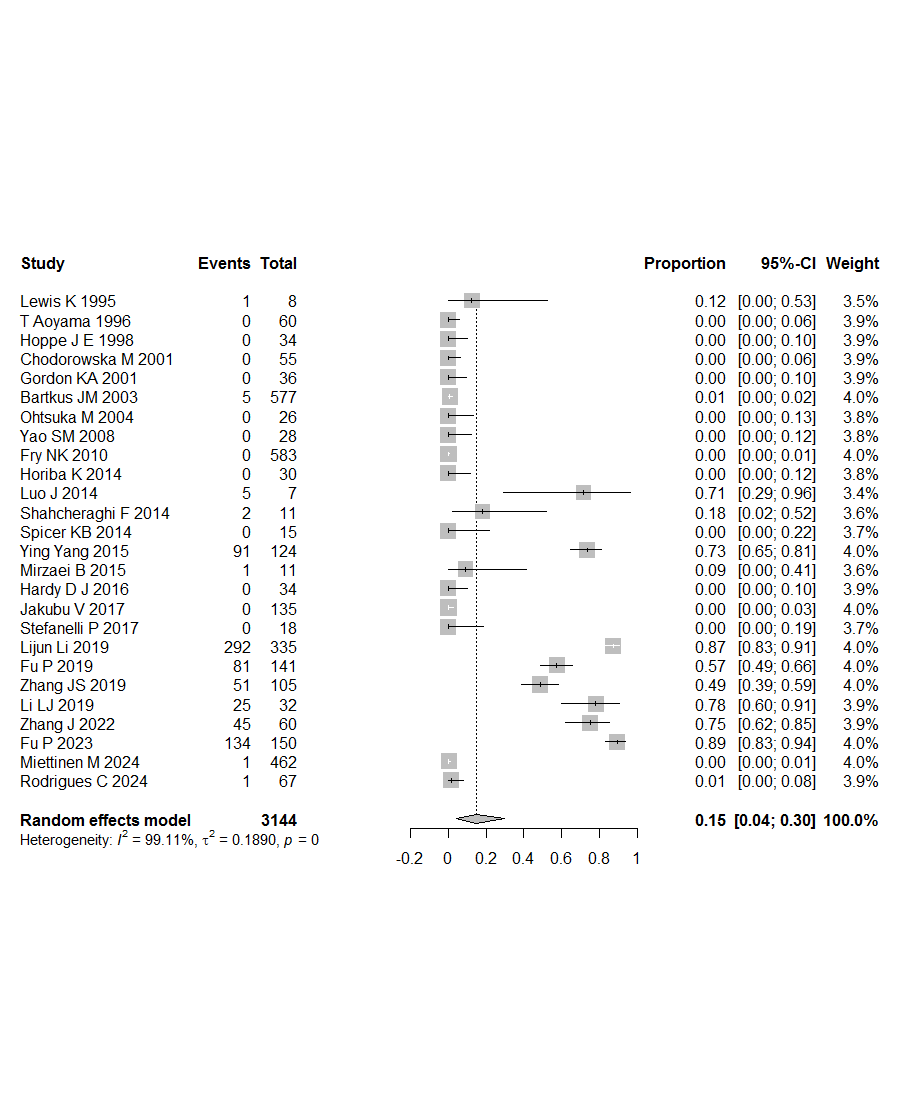


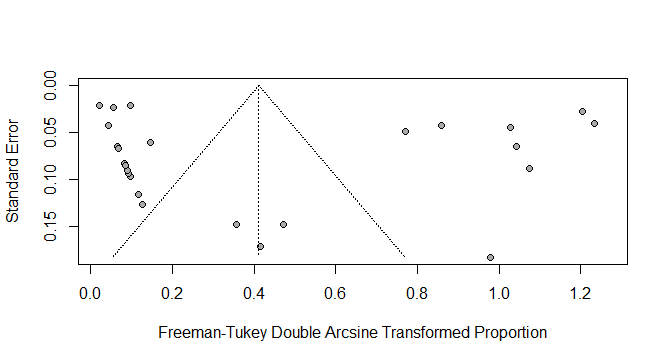


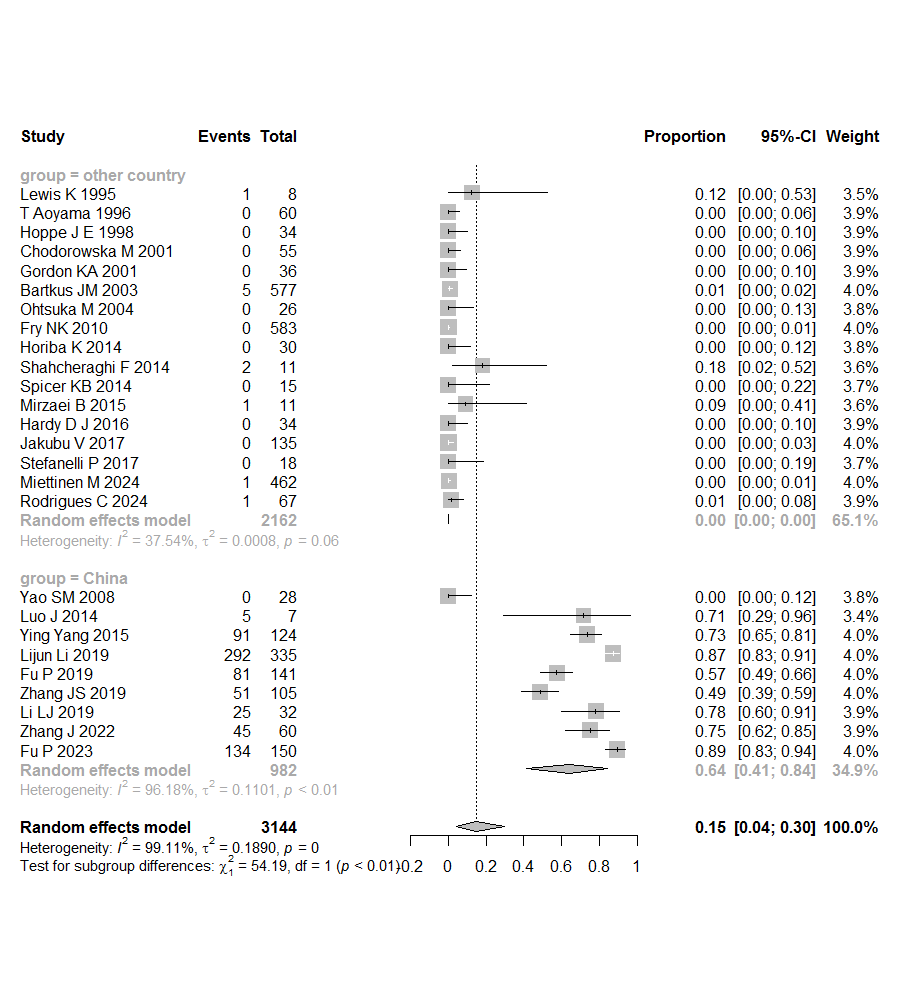


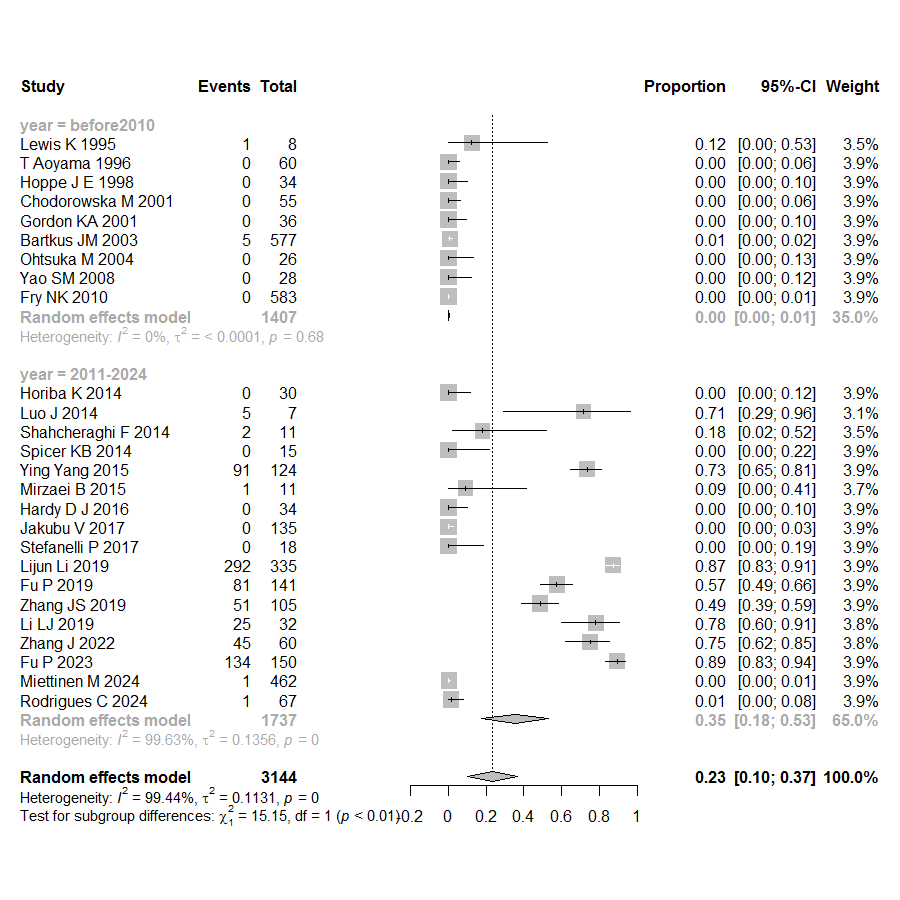


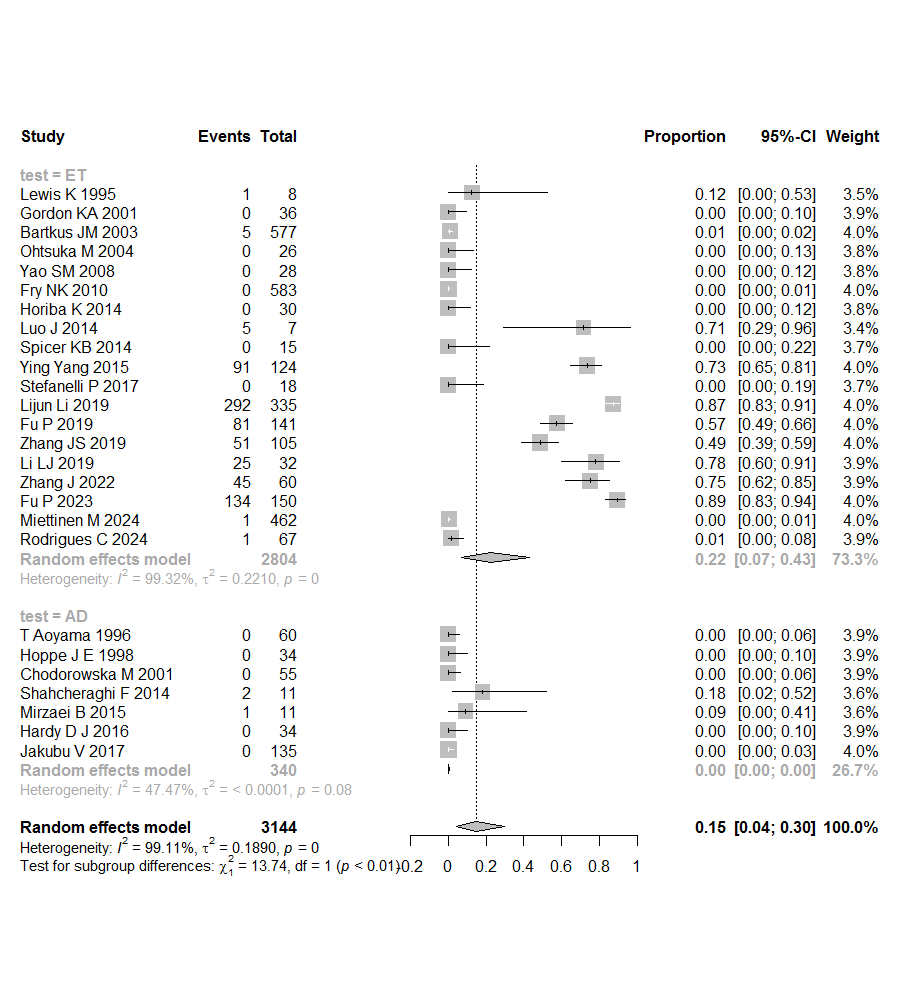


**Clindamycin**

**
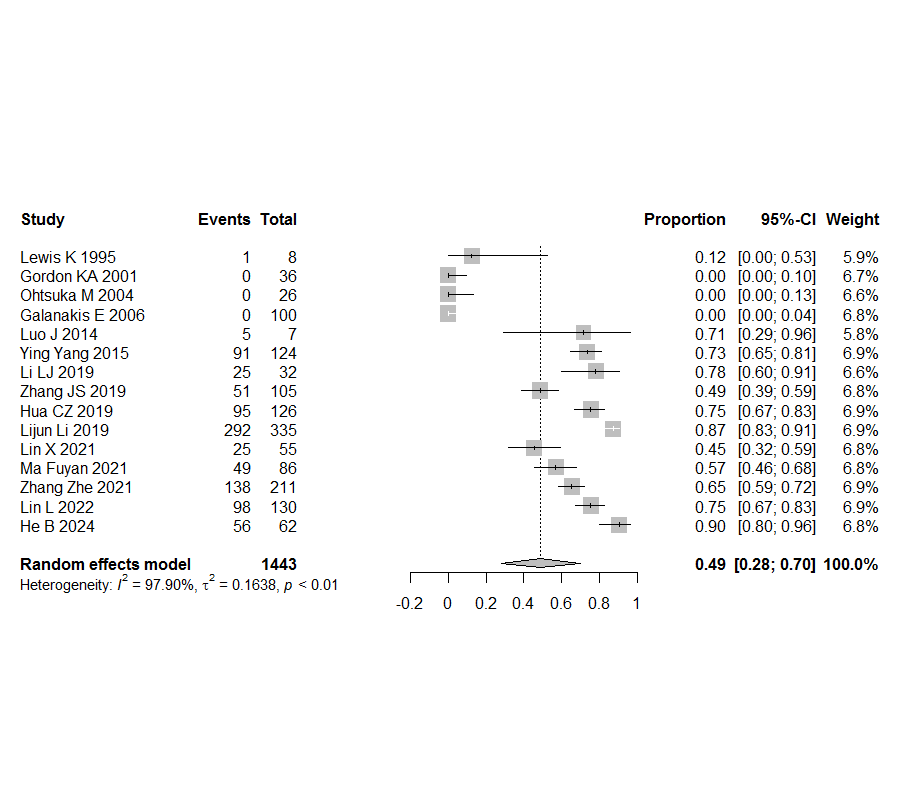
**


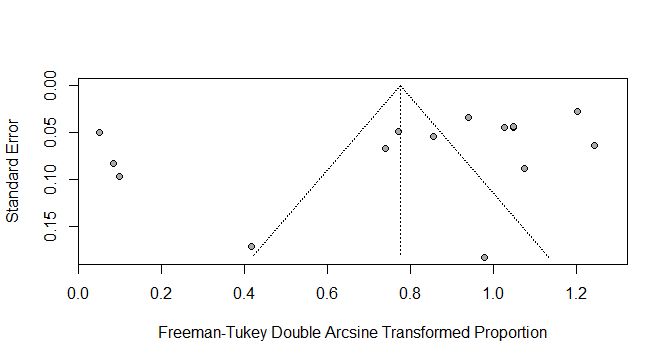


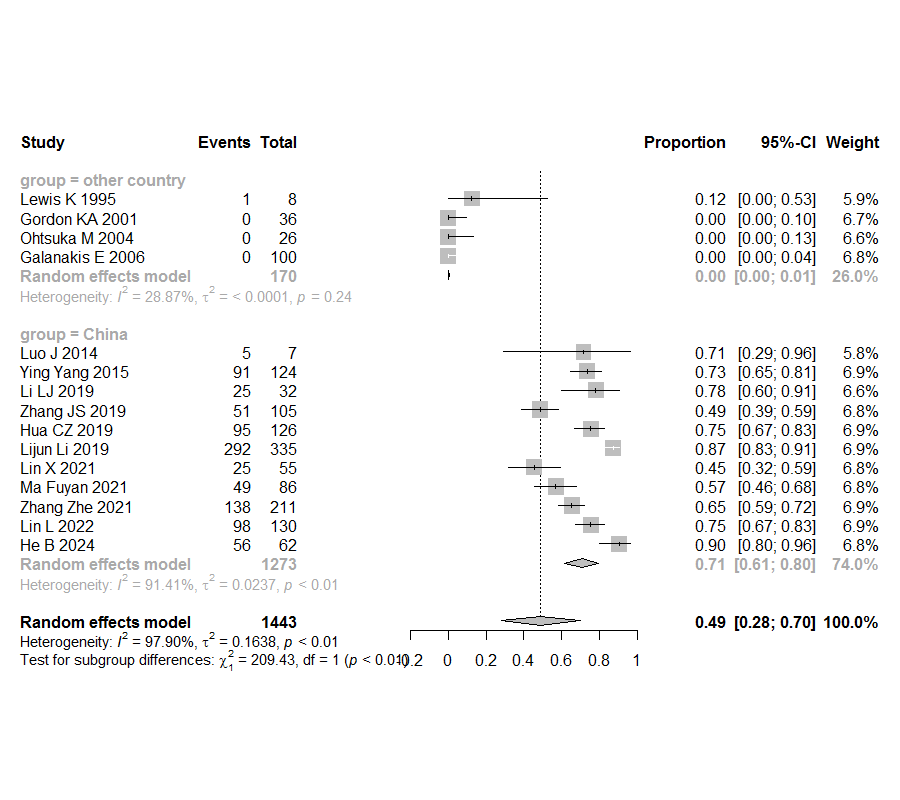


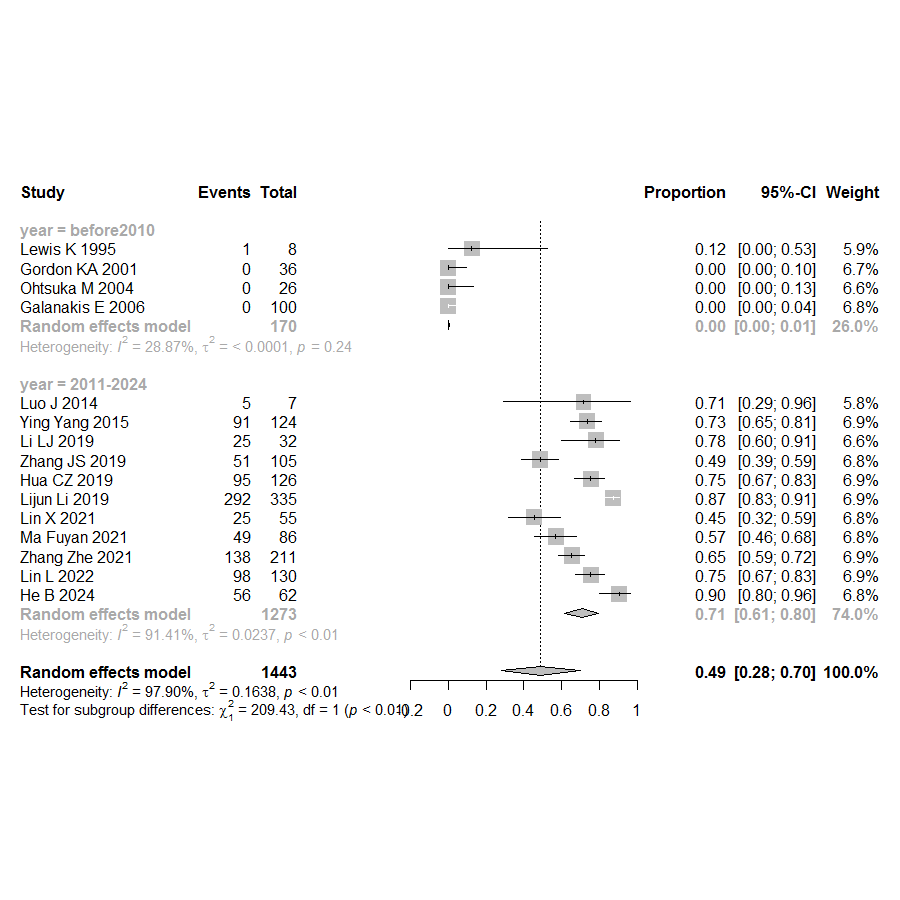

Supplement: Ma et al. supplementary material 2 — Ma et al. supplementary material [file S0950268826101010sup002.docx]
